# Enigmatic incongruence between mtDNA and nDNA revealed by multi-locus phylogenomic analyses in freshwater snails

**DOI:** 10.1038/s41598-019-42682-0

**Published:** 2019-04-17

**Authors:** Takahiro Hirano, Takumi Saito, Yoshihiro Tsunamoto, Joichiro Koseki, Bin Ye, Van Tu Do, Osamu Miura, Yoshihisa Suyama, Satoshi Chiba

**Affiliations:** 1 Department of Biological Sciences, University of Idaho, Moscow, Idaho USA; 2 Graduate school of Life Sciences, Tohoku University, Miyagi, Japan; 3 Kawatabi Field Science Center, Graduate School of Agricultural Science, Tohoku University, Miyagi, Japan; 4 Agricultural Experiment Station, Zhejiang University, Hangzhou, China; 5 Institute of Ecology and Biological Resources, Vietnam Academy of Science and Technology, Hanoi, Vietnam; 6 Faculty of Agriculture and Marine Science, Kochi University, Kochi, Japan; 7 Center for Northeast Asian Studies, Tohoku University, Miyagi, Japan

**Keywords:** Phylogenetics, Taxonomy

## Abstract

Phylogenetic incongruence has frequently been encountered among different molecular markers. Recent progress in molecular phylogenomics has provided detailed and important information for evolutionary biology and taxonomy. Here we focused on the freshwater viviparid snails (*Cipangopaludina chinensis chinensis* and *C. c. laeta*) of East Asia. We conducted phylogenetic analyses and divergence time estimation using two mitochondrial markers. We also performed population genetic analyses using genome-wide SNPs. We investigated how and which phylogenetic patterns reflect shell morphology. The results showed these two species could be separated into four major mitochondrial clades, whereas the nuclear clusters supported two groups. The phylogenetic patterns of both mtDNA and nDNA largely reflected the geographical distribution. Shell morphology reflected the phylogenetic clusters based on nDNA. The findings also showed these two species diversified in the Pliocene to early Pleistocene era, and occurred introgressive hybridisation. The results also raise the taxonomic issue of the two species.

## Introduction

Molecular phylogeny provides a robust framework for investigations in the fields of taxonomy and conservation biology, such as on species diversity and the patterns of geographical distribution^1^. For example, molecular phylogenetic studies have clarified the taxonomic issues of whether species are endemic or alien^2^ and the existence of cryptic species^3^. However, by comparing the phylogenetic patterns determined using different molecular markers, it has become clear that there is incongruence between the molecular markers even in a lineage determined using the same sample^4,5^. To resolve this problem, the inference of species trees from multi-locus data can be beneficial^6,7^. Although molecular phylogenomics such as a multi-locus approach provides more detailed information than an approach that uses a single or a few genes for applications in evolutionary biology and taxonomy^8–10^, such study is still limited.

Under these circumstances, molluscs are an excellent model to compare the relationships among phylogeny, morphology, and geographical distribution patterns. They are the second most diverse phylum of animals^11^ and molluscs also show a high level of local adaptation and genetic divergence among populations^12^. In particular, many studies of freshwater snails have documented substantial incongruence between their molecular phylogeny and morphology, suggesting parallel or convergent evolution^13,14^ along with cryptic speciation^15^, cryptic invasions^16^, and the retention of ancestral polymorphisms or introgression^14^. However, there are not many phylogenetic studies of molluscs using genome wide screens with SNPs so far, therefore, studies investigating mechanisms of incongruence using multi-locus data are needed.

Here, we focus on the freshwater viviparid snails of Japan. *Cipangopaludina chinesis* has two subspecies, *C. c. chinensis* (Gray, 1834) and *C. c. laeta* (Martens, 1860). The taxon *chinensis* is distributed in parts of mainland Asia such as China and Vietnam, and has not been recorded from Japan. On the other hand, *laeta* is distributed in Japan, and was listed as vulnerable due to the declining of populations in the Red Data Book 2014, Threatened Wildlife of Japan^17^. These species have also been spread unintentionally through the expansion of agriculture, such as through rice farming^18^. Therefore, in a previous study it was suggested that *C. c. laeta* is not native to Japan and was actually introduced from China, as these species are not present in the Pleistocene and Holocene fossil record of Japan^18,19^. However, in another study, a fossil was potentially identified as *C. c. laeta* from a Toyono formation (0.5–0.3 Ma^20^). In addition, our previous phylogenetic study showed that *C. c. laeta* of mainland Japan is most likely native because of its genetic differences from nominotypical subspecies *C. c. chinensis* according to GenBank data^21^. However, no comprehensive studies have been performed to clarify the genetic divergence between each population of *C. c. laeta* in the Japanese Archipelago, including Kyushu and the Ryukyu Islands. In particular, because the Kingdom of the Ryukyus frequently undertook cultural exchange with China and mainland Japan, the population of the Ryukyu Islands probably consists of alien populations from mainland Asia and/or mainland Japan.

In the present study, we thus investigate the phylogenetic relationships of the East Asian *C. c. chinesis*/*laeta* subspecies group using mitochondrial and multi-locus nuclear DNA by Sanger and next-generation sequencing. We compare the phylogenetic relationships of mtDNA and nDNA, and clarify whether they are incongruent. We also conduct estimations of divergence time using mtDNA and nDNA. In addition, by using quantitative shell morphological analysis of these species groups, we test which of the phylogenies of mtDNA and nDNA reflect the shell characteristics. Finally, we discuss the observed evolutionary patterns and provide information about these taxa that is of fundamental importance for their taxonomic classification.

## Results

### mtDNA phylogeny

The results of maximum likelihood (ML) and Bayesian analyses were largely consistent with the relatively well-supported clades. Only clades with high support (i.e. posterior probability ≥0.95 or bootstrap support >70%) are here considered further.

**Figure 1:**
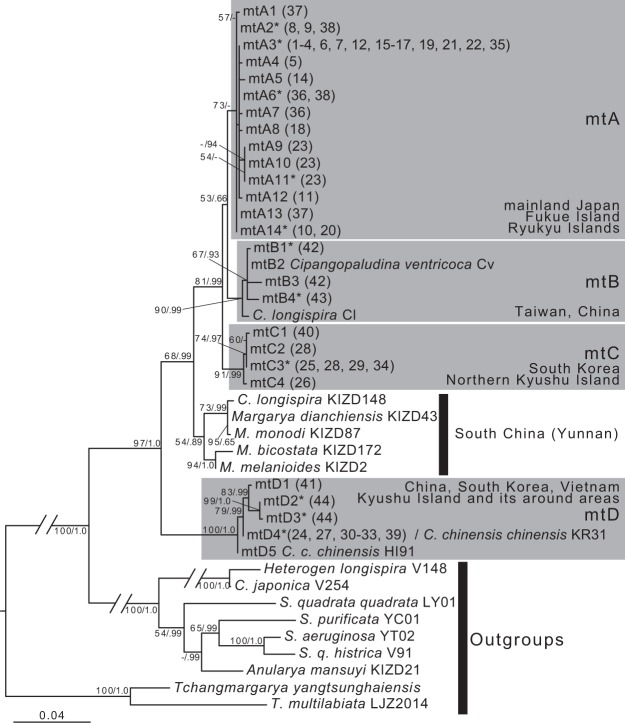
Maximum likelihood consensus tree of East Asian viviparids based on combined sequences from the 16S and COI genes. Each operational taxonomic unit (OTU) label represents the haplotype number in Table S1. Samples from GenBank are indicated each species/subspecies name followed by the specimen ID. The numbers in brackets next to the specimen ID indicate the sampling site number in Fig. 2. Each clade was differentiated by a grey colour. Numbers on branches indicate maximum likelihood bootstrap values followed by Bayesian posterior probabilities. Scale bar indicates 0.04 substitutions per site. The asterisk next to the OTU label indicates a haplotype shared by some other individuals.

**Figure 2:**
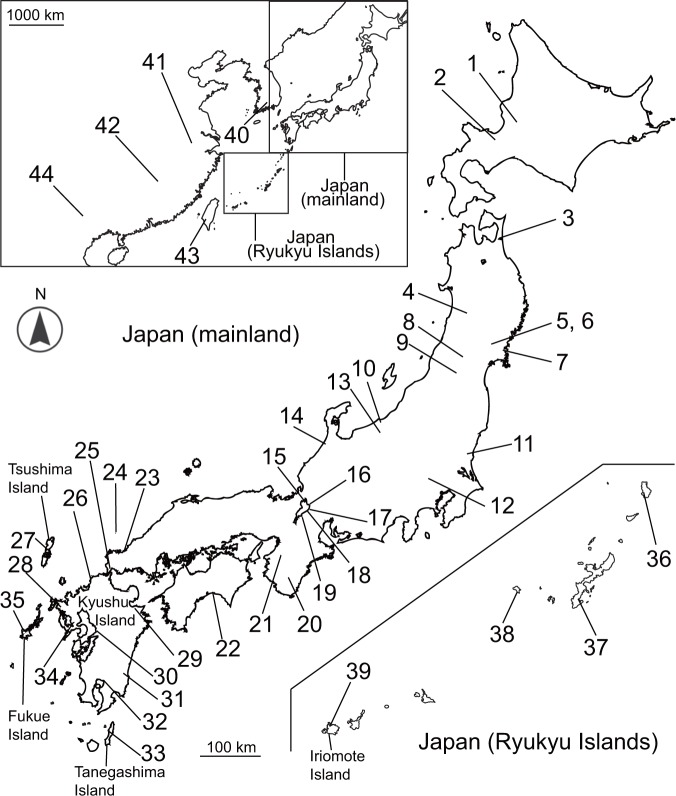
Map showing the sampling sites. Site numbers 1–39 are in Japan including the Ryukyu Islands; site 40 is in South Korea; 41 and 42 are in China; 43 is in Taiwan; and 44 is in Vietnam. See Table S1 for details. The map was created using the software Adobe Illustrator CS6, Macintosh version, (https://www.adobe.com/jp/) and “Map data: Google, DigitalGlobe”.

*Cipangopaludina chinensis* species were separated into four major clades (Fig. 1). Clade mtA was mainly composed of *C. c. laeta* from mainland Japan, though this clade was highly support by ML analysis only. However, we treat this group as monophyletic for convenience. It also included *C. c. laeta* of the Ryukyu Islands, except for Iriomote Island and Fukue Island. The subspecies *C. c. chinensis* of Taiwan and China (Hunan), and two different species [*C. longispira* (Cl) and *C. ventricosa*] formed the clade mtB. In clade mtC, some populations of *C. c. laeta* from the northern part of Kyushu and *C. c. chinensis* of South Korea (Suncheon) were recovered as monophyletic. We recovered sister relationship between clades mtA–C and lineages of south Chinese species [including endemic species of ancient lake groups of Yunnan Province (*Margarya*) and *C. longispira* (KIZD148)]. However, some other populations of Kyushu, Vietnam, South Korea (Haenam), and Hawaii (introduced) clustered together as clade mtD; in addition, in comparison with the relationship between clades mtA–C and the south Chinese species group, clades mtA–C and clade mtD were more genetically different. As a consequence, the phylogenetic relationships reflected the geographical distribution patterns (Fig. 2). Besides, clade mtD was divided into four subclades reflecting the geographical distribution, but the Japanese populations (Kyushu Island) of this clade had only one haplotype (mtD4; Table S1). In contrast, clade mtA was distributed over a wide area but did not have any subclade reflecting the geographical distribution pattern. Notably, a single haplotype (mtA3) was distributed from Hokkaido to Fukue Island.

### Estimation of divergence time using mtDNA

**Figure 3:**
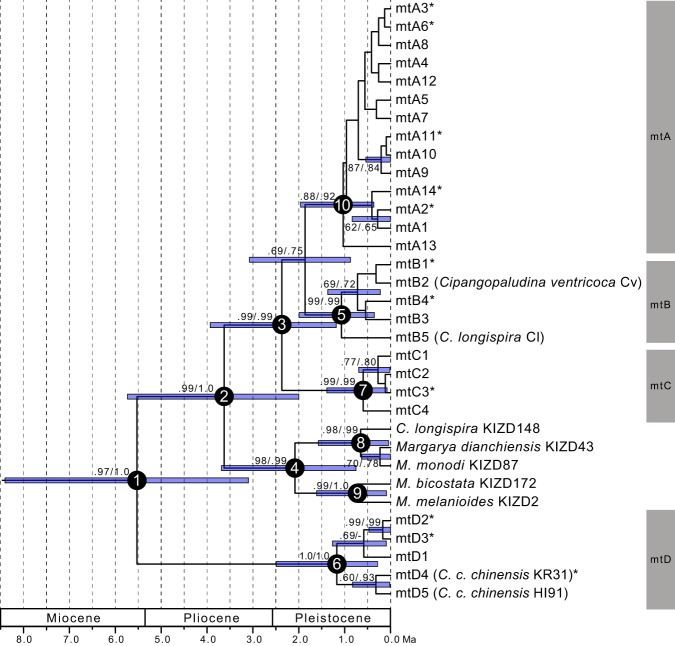
Maximum clade credibility tree generated with the BEAST2 analysis from the combined sequences from the 16S and COI genes. The outgroups (*Anularya*, *Cipangopaludina japonica*, *Heterogen*, *Sinotaia*, and *Tchangmargarya*) are not shown. Each operational taxonomic unit (OTU) label represents the haplotype number in Table S1. Samples from GenBank are indicated each species/subspecies name followed by the specimen ID. Each clade was differentiated by a grey colour. Numbers on branches indicate Bayesian posterior probabilities of UL model followed by Bayesian posterior probabilities of SC model. Node bars indicate a 95% CI for the divergence times by UL model analysis. The principal nodes are named with nominal numbers. The asterisk next to the OTU label indicates a haplotype shared by some other individuals.

For the molecular clock analysis, the ESS values produced in Tracer v.1.6 were considerably higher than 200. Each marginal likelihood estimate is UL model (−3299.3077) and SC model (−3313.7642), and the Bayes factor is 14.4566. For convenience, we first assigned numbers to the cardinal nodes from 1 to 10 (Table 1, Fig. 3). The topology of the tree almost completely coincided with that obtained for the Bayesian tree using MrBayes and the ML analyses (Fig. 1). Positive Bayes factor indicated UL model fit the data best. The mean time of the first divergence of the modern *C. chinensis* group (node 1) was estimated to be 5.53 Ma (UL model, lower and upper 95% HPD interval 3.10–8.41 Ma) and 5.33 Ma (SC model, lower and upper 95% HPD interval 3.89–6.90 Ma). In addition, the mean times for Clade mtA–mtC + South China (including *Margarya*) group (node 2) and Clade mtA + mtB + mtC (node 3) were estimated to be 3.63 Ma (UL model, lower and upper 95% HPD interval 2.00–5.73 Ma) and 2.88 Ma (SC model, lower and upper 95% HPD interval 2.05–3.72 Ma), and 2.37 Ma (UL model, lower and upper 95% HPD interval 1.18–3.93 Ma) and 1.74 Ma (SC model, lower and upper 95% HPD interval 1.16–2.33 Ma), respectively. Thus, the last common ancestor of each clade diversified in the Pliocene and Pleistocene eras. Additional information is shown in Table 1.

**Table 1:** Detailed results of divergence time estimation and geographic analyses.

Node No.	Divergence Time using UL (Ma)	Divergence Time using SC (Ma)
	Mean (Lower CI, Upper CI)	Mean (Lower CI, Upper CI)
1	5.53 (3.10, 8.41)	5.33 (3.89, 6.90)
2	3.63 (2.00, 5.73)	2.88 (2.05, 3.72)
3	2.37 (1.18, 3.93)	1.74 (1.16, 2.33)
4	2.08 (0.75, 3.69)	1.59 (0.93, 2.29)
5	1.07 (0.35, 1.99)	0.77 (0.38, 1.21)
6	1.17 (0.28, 2.49)	0.64 (0.30, 1.02)
7	0.60 (0.08, 1.39)	0.33 (0.09, 0.61)
8	0.65 (0.04, 1.57)	0.46 (0.05, 1.04)
9	0.72 (0.09, 1.61)	0.57 (0.19, 0.99)
10	1.03 (0.36, 1.96)	0.67 (0.31, 0.96)

### Genetic differences of each population using nDNA

**Figure 4:**
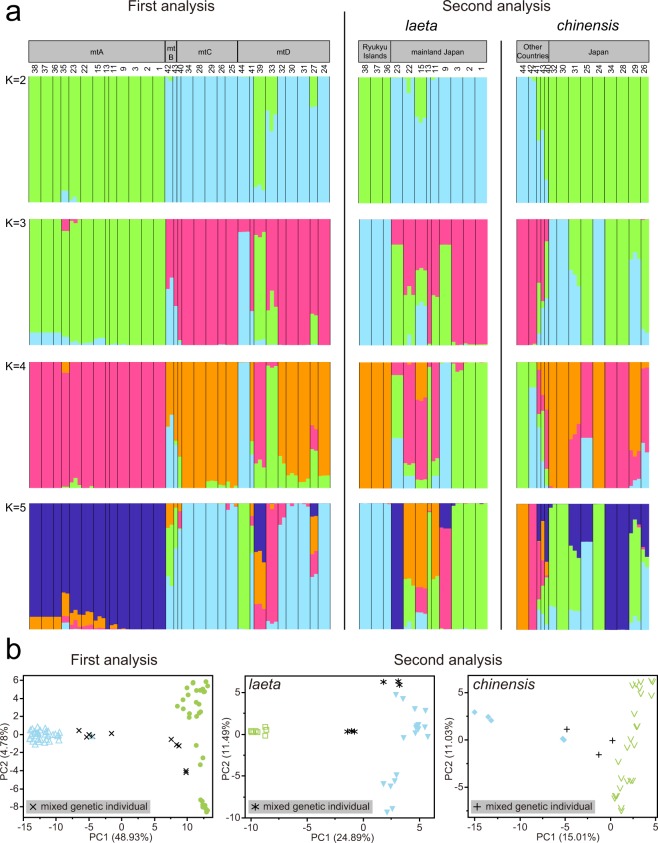
Results of the population genetic analyses. (**a**) Results of the STRUCTURE analyses for K = 2–5. The grey bars and numbers above the results of the STRUCTURE indicate each mitochondrial clade and distribution area, and sampling site number in Fig. 2, respectively. The green and blue colours indicate the two different genetic clusters. (**b**) Plots of results from the principal component analyses (PCA) based on MIG-seq SNPs. The green and blue, and grey symbols under the plots indicate the two genetic clusters and the mixed genetic individuals of STRUCTURE for K = 2, respectively.

Estimated likelihood [LnP (D)] was found to be greatest when K = 2, suggesting that the four mitochondrial clades can be divided into two major clusters of SNPs in the first STRUCTURE (Fig. 4a). These two clusters reflect clades mtA and mtB–D. However, the lineage of MIG-seq SNP was not divided corresponding to the mitochondrial clades mtB–D, even when comparing hierarchically from K = 2 to K = 5. In addition, at least in four populations from the islands adjacent to Kyushu Island (Tsushima Island: Loc. No. 27, Tanegashima Island: 33, and Fukue Island: 35) and Iriomote Island (39), there are mixed genetic structures between the two major clusters. A scatter plot of the first PCA also showed that these specimens can be divided into two major clusters reflecting the result of the first STRUCTURE (Fig. 4b). The four populations, which have the mixed genetic structure as revealed by the first STRUCTURE, showed positions relatively intermediate between those of the two major clusters in the plot. These results indicated that the two major genetic clusters could be regarded as *C. c. laeta* and *C. c. chinensis*. Therefore, for convenience, we treated these two clusters of MIG-seq SNP as *laeta* and *chinensis* in the second analyses. In the second STRUCTURE analysis for *C. c. laeta* and *C. c. chinensis*, each estimated likelihood [LnP (D)] was also found to be greatest when K = 2 (Fig. 4a). Two SNP clusters of *C. c. laeta* reflect the geographical distribution (one from the Ryukyu Islands, the other from mainland Japan except for Kyushu Island). The populations of the Ryukyu Islands in particular were clearly different from other clusters, even when comparing hierarchically from K = 2 to K = 5. In addition, for the two SNP clusters of *C. c. chinensis*, one includes Japan (Kyushu Island and its around areas), while the second cluster includes other countries, reflecting the geographical distribution. A scatter plot of the second PCA showed that each species is divided into two major groups, in line with the results of the second STRUCTURE (Fig. 4b). Moreover, two populations (Shiga: 15 and Kochi: 22) of *C. c. laeta* and three populations (Suncheon, South Korea: 40, Anhui, China: 41, and Taiwan: 43) of *C. c. chinensis* have a mixed genetic structure in the second STRUCTURE, and were shown to be located in positions relatively intermediate between those of the two major groups in the second PCA plots.

### Results of population demography analysis

**Figure 5:**
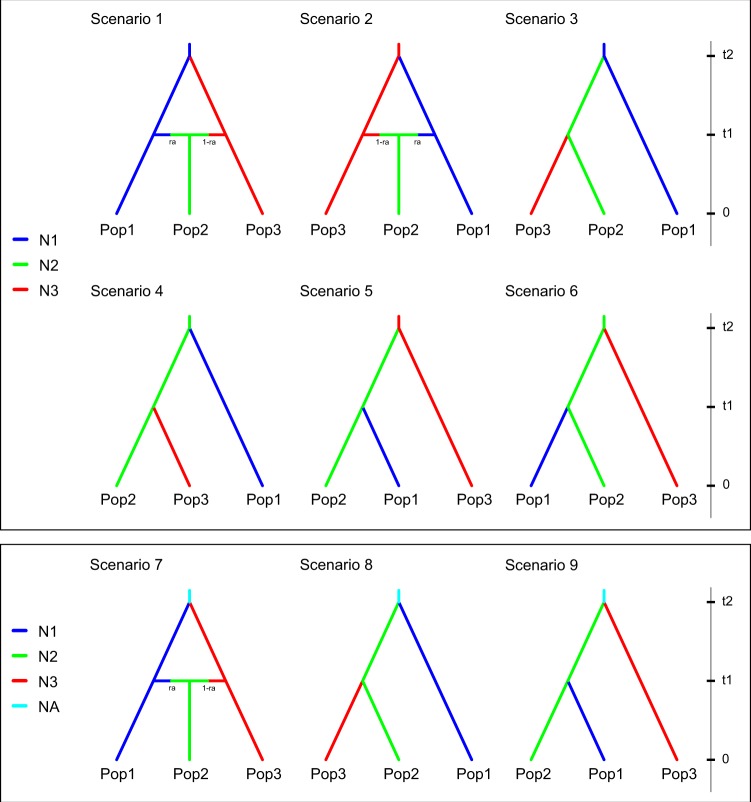
Simulated nine scenarios. Pop1 is Sasebo (V1263–1265), Pop2 is Tanegashima Island (V632, V1416, V1417), and Pop3 is Sapporo (V983, V984). N1, N2 N3 and NA is population size. t1 and t2 is generation time for merging of population.

The results of the population demography analysis by DIYABC using 160 SNPs showed that scenario 7 (Fig. 5) had the highest posterior probability (approximately 0.4) (Fig. S1). Estimated values for each posterior probability of scenarios are listed in Table S2. Based on this scenario, Pop1 and Pop3 initially separated from an unknown ancestral population, and subsequently Pop2 has been composed by Pop1 and Pop3. The estimated parameters of scenario 7 are shown in Table S3. In the case of scenario 7, the median values of divergence times were 2.03 × 10^5^ generations ago for time t1 (95% CI: 4.6 × 10^4^–2.9 × 10^5^) and 5.72 × 10^6^ generations ago for time t2 (95% CI: 1.31 × 10^6^–9.72 × 10^6^) (Fig. S1). Some previous studies suggested that the sexual maturation of ‘*C. chinensis*’ is likely to take one year, with iteroparous reproduction for several years thereafter^22–24^. Therefore *C. c. chinensis* and *C. c. laeta* were separated from the unknown ancestral group before 5,720,000 years ago (t2), and the introgressive hybridisation [the median values of ra: 5.17 × 10^−1^ (95% CI: 3.94 × 10^−1^–6.41 × 10^−1^)] between the two species on Tanegashima Island occurred before 203,000 years ago (t1). The result of PCA from Scenario 7 on model checking option is shown in Fig. S3. In addition, two different ‘hybrid’ scenarios (Scenarios 1 and 2) were chosen as the second and third scenarios (Fig. S1). The type I and type II errors for scenario 7 was 0.554 and 0.436, respectively (Table S4).

### Morphological variation

**Figure 6:**
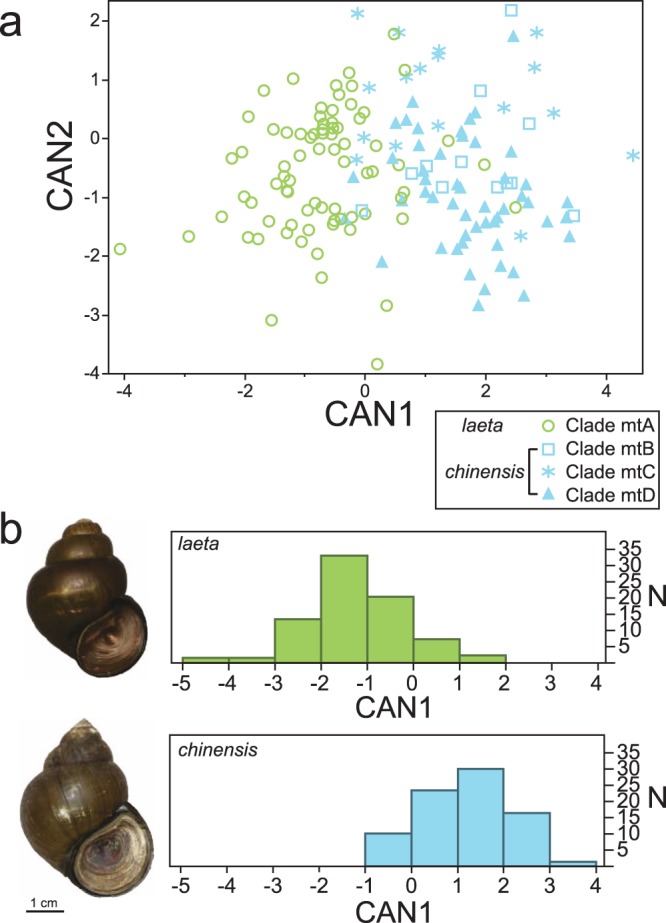
Results of canonical discriminant analysis (CDA). Green and blue indicate *laeta* and *chinensis*, respectively. (**a**) Plots of results from the first CDA based on shell width – D and PCs. The symbols under the plots indicate the taxa of each mitochondrial clade. (**b**) Histogram of results of the second CDA. The photographs on the left indicate each shell morphology. N indicates the number of individuals.

Based on the results of PCA using the Fourier coefficients of all 157 specimens, 11 effective PCs were chosen by SHAPE v1.3 (Table S5). The first CDA was performed based on the 11 PCs and on shell width (Wilks’ lambda = 0.280, F = 6.287, P < 0.001; Fig. 6a; Table S6). The first and second canonical components explained 77.58% and 90.21% of the total variance, respectively. The second CDA was performed based on the same 11 PCs and on shell width (Wilks’ lambda; value = 0.412, F = 17.15, P < 0.001; Fig. 6b; Table S6). The first canonical components explained 100% of the total variance. The first and second CDA results showed that there was little overlap in shell morphology between *C. c. chinensis* and *C. c. laeta*; nevertheless, clades mtB–D largely overlapped.

### Discussion

Enigmatic incongruence between mtDNA and nDNA was found in this study. Two closely related mitochondrial clades (mtB and mtC) and a different clade (mtD) clustered into a monophyletic group in the phylogenetic relationships obtained from the nDNA (Fig. 4). In addition, the shell morphological pattern reflected the phylogenetic relationship of nDNA more than that of the mtDNA (Fig. 6). Therefore, the specimens used in this study can be separated into two different subspecies: *C. c. chinensis* and *C. c. laeta*. In general, for a number of reasons, gene trees sometimes depart from species trees. Explanations for this include the presence of cryptic species^25,26^, incomplete lineage sorting^27^, and introgressive hybridisation^28^. Dealing with the incongruence between genes is a central issue in phylogeny^29^, but the cause of the incongruence in this study is still unclear.

However, our results indicate that the phylogenetic incongruence might have occurred through introgressive hybridisation. The results of divergence time estimation using mtDNA (node 1) are almost included in the timescale of t2 of the ABC analysis, suggesting that these two species diversified in the Pliocene to early Pleistocene era (Fig. 3; Table S3). In addition, the results of DIYABC also indicate that these two subspecies *C. c. chinensis* and *C. c. laeta* actually undertook introgressive hybridisation (t1) after the divergence of clades mtA–C (node 3). According to a previous study, genetic information of extinct species can be included in those of modern species^30^. Therefore, hybridisation with extinct species (or population) occurred, which might have caused the incongruence. As an alternative hypothesis, considering possibility of hybridisation between two extant species, ancient introgressive hybridisation between the last common ancestors of clades mtA–C and mtD may also be the cause of the incongruence. After that ancient introgressive hybridisation, the last common ancestors of clades mtA–C would have been divided into three different clusters by geographical events.

In any case, the mechanism by which specific genotypes spread is unclear. For example, it remains unresolved why the genetic information of the extinct species cannot be found in the nDNA. A possible explanation of this is differences in population size. In addition, non-neutral mtDNA may affect the observed incongruence. mtDNA is related to respiration and temperature adaptations^31–33^, so relatively adaptive mtDNA types for each habitat and environment may remain and spread^34^. In the case of freshwater fish, one mtDNA haplotype spread after introgressive hybridisation^35^. Some phylogenetic studies using freshwater molluscs showed a complex pattern of mtDNA phylogeny^13,21,36^. Taking the above reason into account, the phylogeny of some freshwater organisms is probably associated with whether the mtDNA haplotype is adaptive to each habitat and environment. Moreover, directionality in producing a juvenile may affect the patterns of mtDNA variations. For example, in the case of crossbreeding between female *C. c. laeta* and male *C. c. chinensis*, *C. c. laeta* probably has its juveniles, while in the opposite pattern, *C. c. chinensis* cannot have its juveniles. If directional crossbreeding between the extinct and modern species occurred, it may have resulted in the incongruence shown in this study. In any case, to comprehensively address the question whether introgressive hybridization occurred, and to clarify the role of hybridization in genetic divergence and the mechanism of introgression, additional population genetic analysis using more individuals of these two species and a crossbreeding experiment are needed.

Whatever the cause of the incongruence, the high dispersal ability of these snails may also be related to their unique phylogeny. Notably, each haplotype of mtA, C, and B is distributed across a relatively wide area (Figs 1 and 2; Table S1). The cause of such rapid expansion in their distribution range is unclear, but the geographical distance and phylogenetic relationship of both species do not necessarily coincide, even when considering the trees of nDNA; therefore, some populations may be formed by human activity. In fact, *C. c. chinensis* and/or *C. c. laeta* have been introduced into North America by human activity over the last two centuries^37^, and also into Europe^38^, as well as alien populations becoming established within Japan: a population of Iriomote Island might have been introduced from population of western part of mainland Japan (Yamaguchi Prefecture)^39^. In addition, this genus including *C. c. chinensis* and *C. c. laeta* inhabits various natural and artificial freshwater environments, including shallow lakes, streams, wetlands, and ponds, as well as rice and taro farms^40–42^. Originally, these species inhabited the natural habitat, but in general, these habitats are unstable and the acidity readily increases due to the accumulation of plant remains. Shells are easily decomposed in highly acidic water^43^, so few fossils of these species might have been obtained in Japan.

While these species may have high dispersal ability, *C. c. laeta* of the Ryukyu Islands is genetically different from that of mainland Japan, and is grouped together in an island-specific manner. In clade mtA, only one individual of the Ryukyu Islands (V656) shares a haplotype (mtA2) with that of mainland Japan (Yamagata), but the other individuals do not have a haplotype that is broadly distributed in mainland Japan (Fig. 1). Therefore, our results suggest that the native and endemic populations of *C. c. laeta* may be distributed in the Ryukyu Islands. However, the reason why *C. c. laeta* was not found in mainland Kyushu is unclear. The results of nDNA showed that the populations on the islands adjacent to Kyushu Island (Tanegashima) were composed of hybrids between *C. c. laeta* and *C. c. chinensis* (Fig. 5, scenario 7); therefore, *C. c. laeta* might have been distributed in the mainland of Kyushu. If the populations of *C. c. laeta* on the Ryukyu Islands are native, *C. c. laeta* was once widely distributed in Japan, including on Kyushu Island, but possibly became extinct due to some large-scale disturbance such as a volcanic eruption. In fact, repeated volcanic eruptions are known to have occurred on Kyushu Island or its adjacent islands^44–46^. For example, a bottleneck event followed by population expansion was suggested to have occurred for the Japanese macaque (*Macaca fuscata yakui*) on Yakushima Island^47^. Alternatively, *C. c. chinensis* may have expanded its distribution on the Kyushu mainland and eradicated *C. c. laeta* by interspecific competition. Additional field surveys are needed to obtain a deeper understanding of the distribution patterns of these two species.

The present findings also raised issues about the taxonomy of these species. Before the work of^48^, the taxon ‘*laeta*’ as used in the present study was treated as *Paludina malleata*, but^48^ indicated that the following three species are synonyms of *laeta*: *Paludina malleata* (Reeve, 1863) and *P. abbreviata* (Reeve, 1863), and *Viviparus stelmaphora* (Bourguignat, 1862), described from Japan and China, respectively. In addition, *C. c. laeta* was also determined to be a subspecies of *C. c. chinensis* in the work of^48^. However, the detailed type localities of *C. c. laeta*, *P. malleata*, and *P. abbreviata* were not identified in the descriptions. According to^49^, *C. c. laeta* was described from a specimen or specimens collected by Siebold, who lived in Nagasaki (on the mainland of Kyushu), so the type specimens of *C. c. laeta* are probably *C. c. chinensis*. However, it is difficult to determine whether this ‘*laeta*’ is *C. c. chinensis* as used in this study using only information on the shell. Nevertheless it may be reasonable to treat ‘*laeta*’ of Japan except for Kyushu Island as *C. malleata*, after this study, though the type locality of *P. malleata* is unclear.

In present study, we treated as *chinensis* for continental species, but actually some different species were described in the continental^50^. Different taxa are included in the continental *Cipangopaludina* (e.g. *C. longispira*, *C. ventricosa*) in GenBank. However, taxon name used in the GenBank data may include misidentification, because the two *C. longispira* belong to different clades respectively (Fig. 1). Alternatively, they probably include cryptic species. Similarly, *Cipangopaludina ventricosa* from GenBank can be also treated as misidentification, or it can be a junior synonym of *C. chinensis* if it is not misidentification. Moreover, these taxonomic issues are probably associated with phenotypic plasticity of shell morphology^21^. In any case, the taxonomic issue surroundings *C. chinensis* may involve another continental species. Some phylogenetic studies using continental species were conducted^13,51,52^. However comprehensive phylogenetic relationship among continental species is still unclear. So we need to perform more sampling from China and some countries of mainland East Asia, and phylogenetic analysis using such samples. In particular, further study using SNPs including *Margarya* and its closely related taxa is needed for clarifying the paraphyly of *C. chinensis*.

Our study revealed a complex evolutionary pattern, including phylogenetic incongruence. We clearly identified the occurrence of introgressive hybridisation of Viviparidae for the first time. In addition, we focused on the role of hybridisation in genetic diversification and adaptive radiation^53,54^. In fact, *Margarya* is closely related to *laeta* and *chinensis*^20^ (Fig. 1), which implies the possibility that the genetic divergence of this genus occurred via hybridisation^13^. Combined analyses using both mtDNA and nDNA, and both simple marker and multi-locus approaches, are useful for clarifying the evolutionary history of these viviparid snails.

## Materials and Methods

### Samples

Sampling was performed in the Japanese Archipelago including the Ryukyu Islands, China, Taiwan, and Vietnam. In total, we collected 210 individuals (Fig. 2, Table S1). Since^48^, Japanese subspecies of *C. chinensis* has been treated as a subspecies *C. c. laeta*. Therefore, for convenience, *C. chinensis* collected from Japan was treated as subspecies *laeta*, whereas snails of other countries were nominotypical subspecies *chinensis* in the present study. A fragment of the foot muscle from each individual was stored in 99.5% ethanol for DNA extraction.

We also obtained GenBank data of *C. c. chinensis* (HI91 and KR31). Moreover, we used GenBank data of *Cipangopaludina longispira, Cipangopaludina ventricosa, Margarya bicostata, Margarya dianchiensis, Margarya melanoides*, and *Margarya monodi* as ingroup for mitochondrial phylogenetic analysis because these species are morphologically and/or genetically closely related to the *C. chinensis* group^13,21,50^. Four Asian genera of Viviparidae, *Anularya mansuyi, Cipangopaludina japonica, Heterogen longispira, Tchangmargarya multilabiata, T. yangtsunghaiensis, Sinotaia aeruginosa, Sinotaia purificata, Sinotaia quadrata quadrata*, and *Sinotaia quadrata histrica*, were also obtained from GenBank as outgroups^21^.

### Mitochondrial phylogenetic analysis

Total DNA was extracted in accordance with the work of ^21^. Fragments of the mitochondrial cytochrome oxidase subunit I (COI) and 16S rRNA genes were amplified using the primers LCO1490 (5′-GGTCAACAATCATAAAGATATTGG-3′) and HCO2198 (5′-TAAACTTCAGGGTGACCAAAAAATC-3′)^55^ for COI and 16S-arL (5′-CGCCTGTTTAACAAAAACAT-3′) and 16S-brH (5′-CCGGTCTGAACTCAGATCACGT-3′)^56^ for 16S. PCR reactions were conducted under the conditions described by^21^. PCR products were purified using Exo-SAP-IT (Amersham Biosciences, Little Chalfont, UK). Sequencing was performed using the PCR primers and BigDye^TM^ Terminator Cycle Sequencing Ready Reaction Kit (Applied Biosystems, Foster City, CA, USA), and electrophoresis was carried out using an ABI 3130xl sequencer (Applied Biosystems). The newly generated sequences have been deposited in the GenBank databases (Table S1).

Alignment of the COI sequences was straightforward and required no gaps; 16S sequences were aligned using MUSCLE^57^. GBLOCKS v0.91b^58^ was used to select regions in the aligned sequences that were confidently aligned for analysis (Table S7). After sequence selection, we summarised the same sequence alignments because of node density artefacts^59,60^. Phylogenetic analyses were conducted for the combined data set using maximum likelihood (ML) and Bayesian Inference. For ML and Bayesian analyses, we used Kakusan4-4.0.2011.05.28^61^ to select the appropriate models for sequence evolution (Table S7). Using the selected models, we performed ML analysis in RAxML HPC2^62^. Nodal support for ML analysis was assessed using bootstrap analyses with 1000 replications. Bayesian analysis was performed in MrBayes v3.1.2^63^ using two simultaneous runs, consisting of four simultaneous chains for 30 million generations and sampling the trees every 1000 generations. The first 20% of trees of each run were discarded as burn-in. Both ML and Bayesian analyses were performed at the San Diego Supercomputer Center through the CIPRES Science Gateway^64^.

### Estimation of divergence time

Approximate divergence times were estimated using the two different clock models (strict clock [SC] and uncorrelated log-normal relaxed clock [UL]) as implemented in BEAST 2.4.5^65^ at the San Diego Supercomputer Center through the CIPRES Science Gateway^64^. We used the Bayes factor based on marginal likelihood estimates to compare each clock model^66^. We used the default parameters except for step number (we set 100 steps). Marginal likelihood estimates were obtained using Path Sampler (included in the BEAST package). The model of COI was chosen from models in which the molecular clock rate based on some fossil records of viviparid snails is considered in^67^. A molecular clock rate (uniform prior) ranging from 0.0068 to 0.0118 (substitutions per site and My) was proposed for the COI gene of the family Viviparidae in^67^. We applied the HKY + G model to both COI and 16S sequences, respectively. In addition, we set the parameters of the site models to Gamma category count = 4 and shape = 1.0 as described by^68^. The Yule process was used to model speciation. The Monte Carlo Markov chain was run four times for 30 million generations with sampling every 1000 generations to ensure that the effective sample size (ESS) values were above 1000 for all parameters. In total, we performed 4 runs (both UL and SC). After that, the first 20% of trees of each run were discarded as burn-in, and the remaining trees were combined to produce an ultrametric consensus tree using Logcombiner and Treeannotator v1.5.3 (included in the BEAST software package).

### MIG-seq analysis and SNP detection

Molecular markers of nDNA generally used for phylogenetic analysis, such as 18S rRNA, 28S rRNA, and histone 3 (H3) genes, have slower divergence rates than mtDNA, and are not suitable for clarifying details of the genetic relationship between *C. c. chinensis* and *C. c. laeta* (Hirano *et al*., in prep). Therefore, we obtained genome-wide SNP information of these species groups by multiplexed inter-simple sequence repeat (ISSR) genotyping by sequencing (MIG-seq^69^) from total DNA. MIG-seq is a microsatellite-associated DNA sequencing technique—a type of reduced representation sequencing that includes restriction site-associated DNA sequencing (RAD-seq)^70^. This method is especially effective for low-quality DNA and small quantities of DNA^71^. Preparation of the MIG-seq library was performed under a method modified from^69^ on an Illumina MiSeq Sequencer (Illumina, San Diego, CA, USA) (Tsunamoto *et al*., unpublished), using an MiSeq Reagent Kit v3 (150 cycle; Illumina).

Removal of the primer regions and quality filtering were conducted in accordance with the work of ^69^. SNPs were called using the Stacks software package version 1.41^72^ for further population genetic analyses. We used Stacks with the following parameters: maximum distance between stacks (M) = 1, maximum distance allowed to align secondary reads to primary stacks (N) = 1, and minimum depth option = 10 (-m 10). In addition, we also used a gapped option. We selected only SNPs recorded at a rate of more than 50% among samples for MIG-seq. We also excluded individuals with a high deficiency rate (≥50%) from the analysis, with the exception of an individual from South Korea (M22436). As a results, 1153 SNPs among all populations were detected.

### MIG-seq population genetic analyses

To compare the results of mtDNA with the genetic structure of nDNA, we estimated individual genotypes of nDNA with STRUCTURE v2.3.4^73,74^, based on the MIG-seq data set (Table S1). The number of preassigned genetic clusters (K) was assumed to range from 1 to 5. We performed 10 independent runs for each K value. Each run included 10,000 burn-in iterations and 10,000 iterations. To help determining the optimal K, ∆K was calculated as described by^74^ using Structure harvester web v0.6.94^75^. Bar charts for the proportions of the membership coefficient of each individual in STRUCTURE analysis in 10 runs for each K were summarised using CLUMPP v1.1.2^76^ and visualised in distruct v1.1^77^. In addition, to determine the genetic structure of nDNA, we also conducted a principal component analysis (PCA) with GenoDive v 2.0b27^78^ as well as STRUCTURE analysis. When we performed the first STRUCTURE and PCA, we used 75 specimens (1153 SNPs). Next, we separately conducted the second STRUCTURE and PCA analyses using 33 specimens (997 SNPs) of *chinensis* and 32 specimens (685 SNPs) of *laeta*, respectively, excluding the mixed genetic population between the two species as estimated by the first STRUCTURE described above. This is because we investigate genetic structure within each genetic group determined by the first STRUCTURE and PCA.

To confirm the history of genetic diversification, we used an Approximate Bayesian Computation (ABC) approach in DIYABC v.2.1^79^. ABC provides estimates of demographic and historical parameters and quantitative comparisons of evolutionary scenario^80,81^. On the basis of the structure analysis and PCA analysis on the SNPs, populations were grouped into three groups: Pop1 [*chinensis*: Sasebo (28); V1263–1265], Pop2 [mixed: Tanegashima Island (33); V632, V1416, V1417], and Pop3 [*laeta*: Sapporo (2); V983, V984]. Because the results of STRUCTURE showed that some populations appear to have a mixed genetic structure between two different genetic groups, the above populations (Pop1–3) were used to investigate how the genetic structure was created. Considering isolation among the groups and an unknown ancestral group (NA), nine scenarios were assumed, as shown in Fig. 5. We conducted the pilot run with wide extensive parameters and all summary statistics values. We used adopted the following parameters based on the results from the pilot run: maximum effective numbers of population size N1 (population size of Pop1) and N3 (population size of Pop3), and of N2 (population size of Pop2) were set to 500,000 and 100,000, respectively. In addition, NA (population size of PopNA) was set to 5,000,000. Maximum effective number of generations was set to 300,000 (t1: time when the newer event occurred) and 10,000,000 (t2: time when the older event occurred). The parameter settings are listed in Tables S8. In total, we used 6 summary statistics (each Mean of non-zero values and Variance of non-zero values of Genetic diversities, Fst distances, and Nei’s distances). We compared the different scenarios by calculating their relative posterior probabilities using a logistic regression method from the 1% of simulated data sets most closely resembling the observed data set. We used the option ‘confidence in scenario choice’ in DIYABC to evaluate the validity in scenario choice. We calculated each scenario specific prior based error using simulated 500 datasets per scenario with the same parameter setting. Under the most likely scenario as determined by the logit transformation of parameters, the posterior distributions of parameters were estimated on the 1% of simulated data sets most closely resembling the observed data set. We also used the option ‘model checking’ with principal component analysis (PCA) using DIYABC to assess the goodness-of-fit of the three scenarios. This option can be used to evaluate the consistency of the observed data with the posterior predictive distribution of the model for the best scenario.

### Morphological analysis

**Figure 7:**
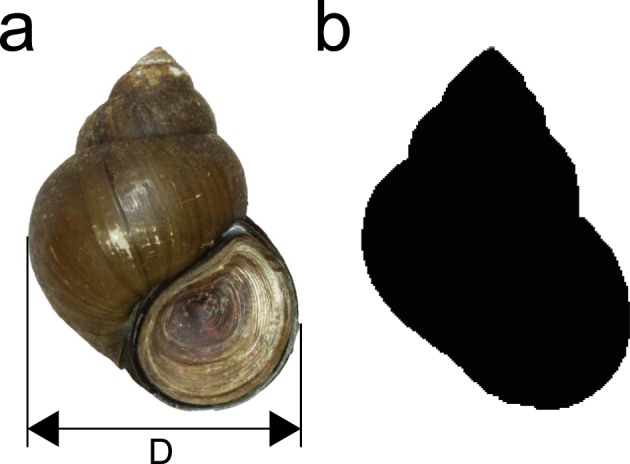
Examples of figure data for the morphological analysis. (**a**) Shell morphology and shell width (D). (**b**) The entire outline of the shell.

To determine whether the phylogenetic relationships of mtDNA and nDNA among the *chinensis*/*laeta* subspecies correspond with the differences in shell morphology, we analysed the variation in shell shape, along the entire outline of the shell, except for the inside aperture and suture, as well as shell width (D; Fig. 7). We used adult shells that have strongly thickened outer lips. In total, we undertook the analyses on 157 specimens (80 individuals of *C. c. chinensis* and 77 individuals of *C. c. laeta*), excluding the mixed genetic population between the two species as estimated by the first STRUCTURE described above.

First, we took a photograph of the shell of each specimen obtained from the field and measured the shell width using digital caliper (Tables S1 and S5). For the quantitative evaluation of entire shell shape, we generated elliptic Fourier descriptors (EFDs^82^) using the same digital images taken by us. All of the images used for the morphological analysis were placed so as to face the shell aperture forward. The parameter of harmonic amplitudes was set to n = 40. For the analysis of shell shape using Fourier coefficients obtained from the EFDs, we conducted a principal component analysis (PCA). We used SHAPE v1.3 to process the digital images, obtain EFDs, and perform PCA^83^.

Second, we conducted a canonical discriminant analysis (CDA) based on PCs obtained from PCA using the EFDs and shell width. The number of effective PCs was calculated as the number of PCs that account for a proportion of the variability larger than 1/number of PCs analysed using SHAPE ver. 1.3. The CDAs were conducted with XLSTAT (Addinsoft, Paris, France). When we performed the first CDA, these scores were compared for each mtDNA clade. Next, to investigate the relationship between the lineage of nDNA (MIG-seq SNPs) and shell morphology, we conducted a second CDA.

## Supplementary information

Supplementary info
