# Supplementary material for: Enigmatic incongruence between mtDNA and nDNA revealed by multi-locus phylogenomic analyses in freshwater snails
Source: Sci Rep. 2019 Apr 17;9:6223. doi: 10.1038/s41598-019-42682-0 (PMC6470147; doi:10.1038/s41598-019-42682-0)
Supplement: Supplementary file 1 — Supplementary info [file 41598_2019_42682_MOESM1_ESM.pdf]

Supplementary Information

Enigmatic incongruence between mtDNA and nDNA revealed by multi-locus phylogenomic analyses in freshwater snails

Takahiro Hirano[1*], Takumi Saito[2], Yoshihiro Tsunamoto[3], Joichiro Koseki[2], Bin Ye[2,4], Van Tu Do[5], Osamu Miura[6], Yoshihisa Suyama[3] & Satoshi Chiba[2,7]

[1]*Department of Biological Sciences, University of Idaho, Moscow, Idaho, USA*

[2]*Graduate school of Life Sciences, Tohoku University, Miyagi, Japan*

[3]*Kawatabi Field Science Center, Graduate School of Agricultural Science, Tohoku University, Miyagi, Japan*

[4]*Agricultural Experiment Station, Zhejiang University, Hangzhou, China*

[5]*Institute of Ecology and Biological Resources, Vietnam Academy of Science and Technology, Hanoi, Vietnam*

[6]*Faculty of Agriculture and Marine Science, Kochi University, Kochi, Japan*

[7]*Center for Northeast Asian Studies, Tohoku University, Miyagi, Japan*

Correspondence and requests for materials should be addressed to T. H.
(email: hirano0223t@yahoo.co.jp)

Table S1. Sampling localities (see Fig. 2), number of individuals used in phylogenetic and morphological analyses and GenBank accession number of each DNA sequences.

Taxa	Specimen ID	Locality name	Site no	16S	COI	mtDNA haplotype	MIG-seq SNP groups	Shell
**Ingroup**								
Cipangopaludina								
chinensis chinensis	M22436	Suncheon, South Korea	40	LC028473	LC028537	mtC1	*chinensis*	
	V766	Lang Son, Vietnam	44	LC437685	LC437749	mtD2	*chinensis*	*
	V767			LC437686	LC437750	mtD2	*chinensis*	*
	V768			LC437687	LC437751	mtD2	*chinensis*	
	V769							*
	V771							*
	V817			LC437688	LC437752	mtD3		
	V818			LC437689	LC437753	mtD3		
	V826							*
	V850	Hunan, China	42	LC437690	LC437754	mtB1	*chinensis*	*
	V851			LC437691	LC437755	mtB1	*chinensis*	*
	V852			LC437692	LC437756	mtB1		*
	V853			LC437693	LC437757	mtB3		*
	V854			LC437694	LC437758	mtB1		
	V1173	Tainan, Taiwan	43	LC437695	LC437759	mtB4		*

	V1174			LC437696	LC437760	mtB4	*chinensis*	*
	V1175							*
	V1176							*
	V1177							*
	V1178							*
	V1179							*
	V1180							*
	V1368	Anhui, China	41	LC437697	LC437761	mtD1	*chinensis*	*
	HI91	Hawaii, USA (introduced)		FJ710214	FJ710298	mtD5		
	KR31	Haenam, South Korea		FJ710213	EU528474	mtD4		
C. c. laeta	V15	Maibara, Shiga, Japan	17	LC028474	LC028538	mtA3		
	V18	Kurihara, Miyagi, Japan	5	LC028476	LC028540	mtA4		
	V32	Bibai, Hokkaido, Japan	1	LC028472	LC028536	mtA3	*laeta*	*
	V33						*laeta*	*
	V34						*laeta*	*
	V35							*
	V170	Kurihara (2), Miyagi, Japan	6	LC028475	LC028539	mtA3		
	V172	Totsukawa, Nara, Japan	20	LC437698		mtA14		

V173			LC437699		mtA14		
V215	Nishiasai, Nagahama, Shiga, Japan	15	LC028477	LC028541	mtA3	*laeta*	
V216						*laeta*	
V217						*laeta*	*
V218							*
V249	Hikone, Shiga, Japan	18	LC028478	LC028542	mtA8		
V343	Kahoku, Yamagata, Japan	8	LC028479	LC028543	mtA2		
V344	Minamisanriku, Miyagi, Japan	7	LC028484	LC028548	mtA3		
V365	Omihachiman, Shiga, Japan	19	LC028480	LC028544	mtA3		
V384	Nagahama, Shiga, Japan	16	LC028481	LC028545	mtA3		
V393	Kahoku, Ishikawa, Japan	14	LC028482	LC028546	mtA5		
V410	Iwade, Wakayama, Japan	21	LC028483	LC028547	mtA3		
V449	Shinano, Nagano, Japan	13				*laeta*	

V450							*
V451							*
V452							*
V454							*
V477	Isahaya, Nagasaki, Japan	34				*chinensis*	
V478						*chinensis*	
V479						*chinensis*	
V480							*
V481			LC437700	LC437762	mtC3		*
V530	Nanyo, Yamagata, Japan	9	LC437701	LC437763	mtA2	*laeta*	*
V531			LC437702	LC437764	mtA2	*laeta*	*
V532			LC437703	LC437765	mtA2	*laeta*	*
V533							*
V535							*
V536							*
V537							*
V538							*
V540							*
V552	Naka, Ibaraki, Japan	11					*

V553			LC437704	LC437766	mtA12	*laeta*	
V554						*laeta*	*
V555							*
V556							*
V557							*
V558							*
V559							*
V560							*
V561							*
V562							*
V563							*
V564							*
V565							*
V566							*
V567							*
V568							*
V571							*
V572							*
V573							*
V632	(Tanegashima Island), Nishinoomote,	33	LC437705	LC437767	mtD4	mixed	

	Kagoshima, Japan						
V1416						mixed	
V1417						mixed	
V653	Nishihara, Okinawa, Japan	37	LC437706	LC437768	mtA1		*
V654			LC437707		mtA13		*
V1161						*laeta*	*
V1162						*laeta*	*
V1163						*laeta*	*
V1164							*
V1165							*
V1166							*
V1167							*
V1168							*
V1169							*
V1170							*
V1171							*
V1172							*
V656	Kume, Okinawa, Japan	38	LC437708	LC437769	mtA2	*laeta*	
V657				LC437770	mtA6	*laeta*	*
V658						*laeta*	*

V659							*
V661							*
V705	Fukuoka, Japan	26	LC437709	LC437771	mtC4	*chinensis*	*
V707						*chinensis*	*
V708							*
V735	(Fukue Island), Goto, Nagasaki, Japan	35	LC437710	LC437772	mtA3	mixed	
V736			LC437711	LC437773	mtA3	mixed	
V737			LC437712	LC437774	mtA3		
V745	(Iriomote Island), Taketomi, Okinawa, Japan	39	LC437713	LC437775	mtD4	mixed	
V746			LC437714	LC437776	mtD4	mixed	
V747			LC437715	LC437777	mtD4	mixed	
V755	Isen, Kagoshima, Japan	36		LC437778	mtA6	*laeta*	*
V756			LC437716	LC437779	mtA7	*laeta*	*
V840	Tohoku, Aomori, Japan	3	LC437717	LC437780	mtA3	*laeta*	*
V841			LC437718	LC437781	mtA3	*laeta*	
V842						*laeta*	*
V843							*
V846							*

V847							*
V855	Mishima, Hagi, Yamaguchi, Japan	24	LC437719	LC437782	mtD4	*chinensis*	*
V856			LC437720	LC437783	mtD4	*chinensis*	*
V857						*chinensis*	*
V858							*
V859							*
V860							*
V861							*
V862							*
V863							*
V864							*
V865							*
V866							*
V867							*
V868							*
V869							*
V870							*
V871							*
V872							*
V873							*

V874							*
V875							*
V983	Sapporo, Hokkaido, Japan	2	LC437721	LC437784	mtA3	*laeta*	*
V984			LC437722	LC437785	mtA3	*laeta*	*
V985						*laeta*	
V986							*
V988							*
V989							*
V1029	Shimonoseki, Yamaguchi, Japan	25	LC437723	LC437786	mtC3	*chinensis*	*
V1030			LC437724	LC437787	mtC3	*chinensis*	*
V1031			LC437725	LC437788	mtC3	*chinensis*	*
V1045	Miyazaki, Miyazaki, Japan	31	LC437726	LC437789	mtD4	*chinensis*	*
V1046			LC437727	LC437790	mtD4	*chinensis*	*
V1047						*chinensis*	*
V1048							*
V1049							*
V1050							*
V1051							*

V1052							*
V1053							*
V1054							*
V1064	Oita, Oita, Japan	29	LC437728	LC437791	mtC3	*chinensis*	
V1065			LC437729	LC437792	mtC3	*chinensis*	*
V1066						*chinensis*	*
V1067							*
V1069							*
V1070							*
V1084	Kirishima, Kagoshima, Japan	32	LC437730	LC437793	mtD4	*chinensis*	*
V1085			LC437731	LC437794	mtD4	*chinensis*	
V1086	Kumamoto, Kumamoto, Japan	30	LC437732	LC437795	mtD4	*chinensis*	*
V1087			LC437733	LC437796	mtD4	*chinensis*	*
V1088						*chinensis*	*
V1089							*
V1090							*
V1091							*
V1092							*
V1093							*

V1094							*
V1095							*
V1096							*
V1097							*
V1098							*
V1154	Yokote, Akita, Japan	4	LC437734	LC437797	mtA3		
V1155			LC437735	LC437798	mtA3		
V1199	Jyoetsu, Niigata, Japan	10	LC437736		mtA14		
V1204			LC437737		mtA14		
V1205	Hagi, Yamaguchi, Japan	23	LC437738		mtA11	*laeta*	*
V1206			LC437739		mtA11	*laeta*	*
V1207				LC437799	mtA9	*laeta*	*
V1208			LC437740	LC437800	mtA10		
V1209							*
V1212							*
V1213							*
V1215							*
V1218	(Tsushima Island), Tsushima, Nagasaki, Japan	27				mixed	

	V1222			LC437741	LC437801	mtD4		
	V1223			LC437742	LC437802	mtD4		
	V1224						mixed	
	V1250	Ranzan, Saitama, Japan	12	LC437743	LC437803	mtA3		
	V1251			LC437744	LC437804	mtA3		
	V1263	Sasebo, Nagasaki, Japan	28	LC437745	LC437805	mtC3	*chinensis*	
	V1264				LC437806	mtC2	*chinensis*	
	V1265			LC437746	LC437807	mtC3	*chinensis*	*
	V1270							*
	V1271							*
	V1272							*
	V1310	Nankoku, Kochi, Japan	22	LC437747	LC437808	mtA3	*laeta*	
	V1311			LC437748	LC437809	mtA3	*laeta*	
	V1312						*laeta*	*
	V1313							*
C. longispira	Cl	Lake Lashihai, Yunnan, China		KJ867106		mtB5		
	KIZD148	Lake Yilong, Yunnan, China		GU198863	GU198809			
C. ventricoca	Cv	Lake Jianhu, Yunnan,		KJ867107		mtB2		

		China		
Margarya bicostata	KIZD172	Lake Dianch, Yunnan, China	GU198866	GU198812
M. dianchiensis	KIZD43	Lake Dianch, Yunnan, China	GU198835	GU198781
M. melanioides	KIZD2	Lake Dianch, Yunnan, China	GU198820	GU198765
M. monodi	KIZD87	Lake Dianch, Yunnan, China	GU198850	GU198796
**Outgroup**				
Anularya mansuyi	KIZD21		GU198823	GU198768
C. japonica	V254		LC028463	LC028527
Hetelogen longispira	V148		LC028455	LC028519
Sinotaia aeruginosa	YT02		JN621302	JN621292
S. purificata	YC01		JN621318	JN621282
S. quadrata quadrata	LY01		JN621313	JN621285
S. q. histrica	V91		LC028441	LC028505
Tchangmargarya multilabiata	LJZ2014			KJ636767
T. yangtsunghaiensis			KJ867124	KJ636773

The asterisks indicate individuals used in morphological analysis.

Table S2. Estimated values for each posterior probability of scenarios in DIYABC.

n	scenario 1	scenario 2	scenario 3	scenario 4	scenario 5	scenario 6	scenario 7	scenario 8	scenario 9
3000	0.2825 [0.2420,0.3231]	0.2769 [0.2372,0.3166]	0.0000 [0.0000,0.0000]	0.0000 [0.0000,0.0000]	0.0000 [0.0000,0.0000]	0.0000 [0.0000,0.0000]	0.4405 [0.3953,0.4858]	0.0000 [0.0000,0.0000]	0.0000 [0.0000,0.0000]
6000	0.2941 [0.2651,0.3232]	0.2877 [0.2593,0.3161]	0.0000 [0.0000,0.0000]	0.0000 [0.0000,0.0000]	0.0000 [0.0000,0.0000]	0.0000 [0.0000,0.0000]	0.4181 [0.3865,0.4497]	0.0000 [0.0000,0.0000]	0.0000 [0.0000,0.0000]
9000	0.2928 [0.2693,0.3163]	0.2896 [0.2665,0.3128]	0.0000 [0.0000,0.0000]	0.0000 [0.0000,0.0000]	0.0000 [0.0000,0.0000]	0.0000 [0.0000,0.0000]	0.4176 [0.3919,0.4432]	0.0000 [0.0000,0.0000]	0.0000 [0.0000,0.0000]
12000	0.2920 [0.2717,0.3122]	0.2869 [0.2670,0.3068]	0.0000 [0.0000,0.0000]	0.0000 [0.0000,0.0000]	0.0000 [0.0000,0.0000]	0.0000 [0.0000,0.0000]	0.4212 [0.3990,0.4434]	0.0000 [0.0000,0.0000]	0.0000 [0.0000,0.0000]
15000	0.2919 [0.2739,0.3099]	0.2842 [0.2666,0.3019]	0.0000 [0.0000,0.0000]	0.0000 [0.0000,0.0000]	0.0000 [0.0000,0.0000]	0.0000 [0.0000,0.0135]	0.4238 [0.4040,0.4437]	0.0000 [0.0000,0.0000]	0.0000 [0.0000,0.0000]
18000	0.2921 [0.2757,0.3085]	0.2826 [0.2666,0.2986]	0.0000 [0.0000,0.0000]	0.0000 [0.0000,0.0000]	0.0000 [0.0000,0.0000]	0.0000 [0.0000,0.0122]	0.4253 [0.4072,0.4434]	0.0000 [0.0000,0.0000]	0.0000 [0.0000,0.0000]
21000	0.2927 [0.2775,0.3078]	0.2821 [0.2673,0.2969]	0.0000 [0.0000,0.0000]	0.0000 [0.0000,0.0000]	0.0000 [0.0000,0.0113]	0.0000 [0.0000,0.0113]	0.4252 [0.4085,0.4419]	0.0000 [0.0000,0.0000]	0.0000 [0.0000,0.0000]
24000	0.2931 [0.2789,0.3072]	0.2819 [0.2681,0.2957]	0.0000 [0.0000,0.0000]	0.0000 [0.0000,0.0000]	0.0000 [0.0000,0.0106]	0.0000 [0.0000,0.0106]	0.4250 [0.4094,0.4406]	0.0000 [0.0000,0.0000]	0.0000 [0.0000,0.0000]
27000	0.2928 [0.2795,0.3061]	0.2819 [0.2689,0.2949]	0.0000 [0.0000,0.0000]	0.0000 [0.0000,0.0000]	0.0000 [0.0000,0.0100]	0.0000 [0.0000,0.0099]	0.4252 [0.4106,0.4399]	0.0000 [0.0000,0.0000]	0.0000 [0.0000,0.0000]
30000	0.2924 [0.2799,0.3050]	0.2825 [0.2702,0.2948]	0.0000 [0.0000,0.0000]	0.0000 [0.0000,0.0000]	0.0000 [0.0000,0.0094]	0.0000 [0.0000,0.0094]	0.4250 [0.4111,0.4389]	0.0000 [0.0000,0.0000]	0.0000 [0.0000,0.0000]

Table S3. The estimated parameters of scenario 7 (logit transformation).

Parameter	Median	95% CI (Lower–Upper)
Effective population size		
N1	3.2×10^{5}	8.81×10^{4}–4.86×10^{5}
N2	6.39×10^{4}	1.33×10^{4}–9.69×10^{4}
N3	2.18×10^{5}	4.72×10^{4}–4.76×10^{5}
NA	2.82×10^{6}	2.02×10^{5}–4.89×10^{6}
Time scale in generations		
t1	2.03×10^{5}	4.6×10^{4}–2.9×10^{5}
t2	5.72×10^{6}	1.31×10^{6}–9.72×10^{6}
Admixture		
ra	5.17×10^{-1}	3.94×10^{-1}–6.41×10^{-1}

Table S4. The detailed results of confidence in the option 'scenario choice' on DIYABC

	Scenario 1	Scenario 2	Scenario 3	Scenario 4	Scenario 5	Scenario 6	Scenario 7	Scenario 8	Scenario 9
D1	0.31	0.268	0.022	0.028	0.018	0.022	0.32	0.012	0
D2	0.256	0.302	0.022	0.024	0.018	0.056	0.318	0.012	0.01
D3	0.01	0.016	0.27	0.28	0	0	0.006	0.418	0
D4	0.006	0.012	0.276	0.334	0	0.004	0.004	0.364	0
D5	0.002	0.01	0	0	0.258	0.328	0.012	0	0.39
D6	0.012	0.018	0.002	0.002	0.294	0.326	0.006	0	0.34
D7	0.144	0.168	0.026	0.012	0.012	0.028	0.564	0.024	0.022
D8	0.008	0.008	0.202	0.108	0	0	0.02	0.654	0
D9	0.002	0	0	0	0.186	0.142	0.014	0	0.656

	Scenario 7
Type-I error	0.554
Type-II eeror	0.436

Table S5. Datasets used in morphological analyses of the shell.

Specimen ID	mtDNA clade	nDNA clade	D	PC1	PC2	PC3	PC4	PC5	PC6	PC7	PC8	PC9	PC10	PC11
V32	mtA	*laeta*	37.81	0.00404	-0.00149	-0.00277	-0.0149	0.00726	-0.000798	0.00918	-0.00222	0.00868	-0.00111	0.00125
V33	mtA	*laeta*	36.93	0.0188	0.0103	-0.0112	-0.00154	0.00266	0.0018	-0.000162	-0.00766	0.00934	-0.000542	0.000939
V34	mtA	*laeta*	39.62	0.0319	0.01	0.00537	0.0044	-0.00903	-0.0166	-0.00457	-0.00277	0.00273	0.00211	-0.000337
V35	mtA	*laeta*	39.13	0.0126	-0.0182	-0.00716	-0.00265	0.00681	0.00433	0.0147	-0.00356	0.00666	-0.00632	-0.0028
V217	mtA	*laeta*	26.81	-0.0283	0.00834	-0.00352	-0.0136	-0.0129	-0.000673	-0.00417	-0.0019	-0.00688	0.00526	0.00699
V218	mtA	*laeta*	25.29	-0.0431	-0.00574	-0.0125	0.012	-0.0127	0.00462	-0.0044	0.00778	-0.00391	0.00146	0.00561
V450	mtA	*laeta*	21.35	-0.0166	0.01	0.00195	-0.0205	-0.00493	0.00309	0.00733	-0.00047	-0.00732	-0.0101	0.00642
V451	mtA	*laeta*	27.19	0.0437	-0.0053	0.00448	-0.00914	-0.0105	-0.00325	0.0108	0.00528	0.00145	0.000578	-0.00834
V452	mtA	*laeta*	25.1	0.0395	0.0105	-0.00958	0.00155	-0.0194	0.00434	0.00346	-0.000502	-0.000957	-0.00795	-0.00214
V454	mtA	*laeta*	24.09	0.034	0.0202	-0.00738	-0.00665	-0.0177	0.00368	0.00528	-0.00573	0.000974	-0.000419	-0.00417
V530	mtA	*laeta*	29.38	0.00795	-0.00612	0.000252	-0.0151	0.0119	0.00186	0.0121	0.00807	0.00592	0.00376	0.00545
V531	mtA	*laeta*	29.48	-0.0356	-0.00902	0.00858	0.012	0.00357	-0.00445	0.00242	-0.0125	-0.00393	0.00318	0.00365
V532	mtA	*laeta*	31.72	0.0462	0.00886	-0.00488	0.0114	-0.0155	-0.00075	0.00711	-0.00343	0.0108	0.00867	0.00437
V533	mtA	*laeta*	35.19	0.0109	-0.00796	-0.0178	-0.0119	-0.00562	0.000198	0.00589	-0.0073	0.00667	0.0000421	0.00117
V535	mtA	*laeta*	28.13	-0.0469	0.00496	-0.00501	-0.000973	0.0105	0.00332	0.00587	-0.00497	-0.00211	0.00159	-0.00384
V536	mtA	*laeta*	29.67	0.0179	0.0177	0.000125	-0.00375	0.00637	0.00309	0.0119	-0.00741	-0.00177	-0.00252	0.000281
V537	mtA	*laeta*	27.48	-0.0246	0.013	0.00449	-0.00477	0.0181	-0.00129	0.00855	-0.00121	0.000358	0.00769	-0.00309
V538	mtA	*laeta*	34.17	-0.000898	-0.00553	-0.0071	0.00909	0.00122	0.000758	-0.0017	0.00507	-0.00778	0.00204	0.00826
V540	mtA	*laeta*	31.15	0.0174	-0.00603	-0.0221	-0.00175	0.00313	0.00151	0.00092	-0.00763	0.0143	0.00451	0.00615
V552	mtA	*laeta*	32.16	-0.00145	-0.0155	0.00278	-0.00483	0.0147	-0.00191	-0.00539	-0.0109	-0.00983	-0.00197	0.00304
V554	mtA	*laeta*	37.18	0.0305	0.000366	-0.0104	-0.0088	-0.00579	-0.0000138	0.0105	-0.011	0.0089	-0.00277	-0.00298
V555	mtA	*laeta*	26.32	-0.0344	-0.00888	0.0055	0.00862	0.0106	0.00112	0.00327	-0.0045	-0.00395	-0.00143	-0.00278
V556	mtA	*laeta*	37.5	-0.0025	-0.0122	-0.017	0.00702	0.00843	0.00105	-0.00253	-0.00106	-0.0153	0.00347	0.00629
V557	mtA	*laeta*	35.35	-0.00126	-0.0165	-0.0299	0.00319	-0.0004	0.00114	0.000354	-0.00552	-0.00797	0.00253	0.00211
V558	mtA	*laeta*	32.52	0.016	-0.0284	-0.00232	0.0113	0.00867	-0.00819	0.00681	-0.00598	-0.00231	-0.00756	0.00235
V559	mtA	*laeta*	33.38	-0.017	-0.000924	-0.00319	-0.00723	0.0127	0.00753	0.00204	-0.00611	-0.0121	-0.000243	0.000959
V560	mtA	*laeta*	32.39	-0.0399	-0.0246	0.0195	0.0163	0.0051	0.0179	0.00295	-0.00796	-0.00686	0.00331	0.00415
V561	mtA	*laeta*	29.93	-0.0198	-0.0238	-0.0115	0.00558	0.00656	-0.00378	0.00623	-0.00484	-0.00875	-0.00481	-0.00194
V562	mtA	*laeta*	24.18	-0.042	0.00884	-0.00823	-0.00322	0.00383	0.00326	0.00114	-0.00089	-0.00259	-0.00244	-0.000927
V563	mtA	*laeta*	24.77	-0.0138	0.015	-0.0112	-0.00771	0.00388	0.00362	0.00744	-0.00445	-0.00628	0.00795	0.00343
V564	mtA	*laeta*	30.94	-0.00648	-0.00611	-0.012	0.00149	0.0106	-0.00068	0.000893	-0.0023	0.00231	-0.00274	-0.000841
V565	mtA	*laeta*	27.16	-0.0274	-0.00199	-0.00092	0.00231	0.00833	0.00202	0.0036	0.001	-0.00544	-0.00381	0.00152
V566	mtA	*laeta*	24.48	-0.0699	-0.00625	0.0188	0.0103	-0.00466	0.0141	0.00651	0.00135	0.00504	-0.00391	0.00486
V567	mtA	*laeta*	22.95	-0.0529	-0.00635	-0.00168	0.00495	-0.00091	0.00848	0.00222	0.00111	0.00019	-0.00263	-0.00458
V568	mtA	*laeta*	23.52	-0.0271	-0.000219	-0.00222	-0.00675	0.0144	0.00582	0.00487	0.000595	-0.00168	-0.00696	-0.00231
V571	mtA	*laeta*	26.78	-0.00851	0.0251	-0.00267	-0.014	0.00325	-0.00529	0.00481	-0.00982	-0.0111	-0.00729	0.0006
V572	mtA	*laeta*	36.24	-0.0335	-0.026	0.00802	0.00954	0.000748	-0.00806	-0.00352	-0.00308	-0.0154	0.00388	0.000363
V573	mtA	*laeta*	36.85	-0.0206	-0.0184	-0.0189	-0.000892	0.00739	0.00637	-0.00708	-0.00206	-0.00685	0.00114	-0.00476
V653	mtA	*laeta*	24.99	-0.0818	-0.00032	0.0132	0.000111	-0.00941	0.0131	0.0123	0.00326	0.00099	-0.00629	-0.00319
V654	mtA	*laeta*	19.45	-0.0834	-0.00328	0.0155	0.00576	-0.0236	0.00693	0.0000113	-0.0000456	0.0105	-0.00342	0.00789
V657	mtA	*laeta*	30.15	-0.00292	0.0247	-0.00996	-0.00015	-0.000683	0.00615	-0.000283	0.000402	0.00303	0.00131	-0.000161
V658	mtA	*laeta*	28.63	-0.0559	0.00473	0.00358	0.00161	-0.00605	-0.00967	0.0068	0.003	-0.00137	0.00296	-0.000377
V659	mtA	*laeta*	28.17	-0.0251	-0.0104	-0.0139	0.00191	-0.000425	0.00348	0.000167	0.00157	-0.00319	0.00487	-0.000186
V661	mtA	*laeta*	31.55	-0.0219	0.032	-0.0139	0.0181	-0.00576	0.00325	0.00891	0.00104	-0.00447	0.0111	0.000808
V755	mtA	*laeta*	32.64	0.0358	-0.0064	-0.00883	0.000246	-0.0105	0.00224	0.000494	-0.00609	0.00647	0.000805	-0.00403
V756	mtA	*laeta*	32.31	0.00363	-0.00482	-0.00221	0.00381	-0.00787	-0.00696	-0.00588	-0.00294	-0.0034	0.00502	0.00341
V840	mtA	*laeta*	32.72	0.106	-0.04	0.0166	-0.00968	-0.015	0.00779	-0.00178	-0.00314	-0.000207	0.0113	-0.00824
V842	mtA	*laeta*	34.09	0.0267	0.00234	0.016	-0.0264	0.00992	0.00353	-0.00204	-0.00941	0.00698	-0.0019	-0.00131
V843	mtA	*laeta*	28.07	0.0402	-0.000644	0.00708	-0.00587	-0.0107	0.0024	0.0116	-0.00414	-0.0019	-0.00279	-0.00384
V846	mtA	*laeta*	39.92	0.0213	-0.0129	-0.0196	0.0026	-0.000671	0.00153	0.00258	-0.00309	0.00428	-0.00587	0.000459
V847	mtA	*laeta*	33.83	0.0203	0.000338	-0.0128	0.00646	0.00484	-0.0189	0.0192	0.00389	0.00835	0.00454	-0.0104
V983	mtA	*laeta*	39.47	0.0224	-0.0113	-0.0349	0.00742	-0.00458	-0.014	0.00562	-0.00232	0.0062	-0.00524	0.00327
V984	mtA	*laeta*	39.93	0.0371	-0.00565	-0.00845	0.00248	-0.00968	-0.00841	0.00475	-0.00994	0.00384	-0.00169	-0.00147
V986	mtA	*laeta*	38.59	0.0447	-0.0191	-0.032	0.0233	0.00188	-0.00539	-0.00172	-0.016	0.00681	-0.0032	0.000667
V988	mtA	*laeta*	35.16	0.0312	-0.00386	-0.0331	0.0115	0.00854	0.0124	0.00382	-0.00601	0.0121	-0.000731	-0.00181
V989	mtA	*laeta*	31.58	-0.0541	-0.0149	-0.00873	-0.000498	0.00394	0.00333	0.00378	-0.00489	0.00599	-0.000487	0.0000869
V1161	mtA	*laeta*	25.54	-0.0586	0.00404	0.00633	-0.0108	-0.0217	0.00731	0.00172	0.0011	-0.000788	0.00118	0.000344
V1162	mtA	*laeta*	25.54	-0.0255	-0.0229	-0.013	-0.00832	0.000553	-0.00171	-0.00818	0.00483	-0.00178	0.00566	-0.00161
V1163	mtA	*laeta*	24.84	-0.0244	0.0112	-0.000083	-0.0072	-0.0124	-0.00225	0.00726	-0.00313	-0.00702	-0.00136	0.000871
V1164	mtA	*laeta*	25.37	-0.0389	-0.00559	-0.0121	-0.0105	-0.00639	0.0114	-0.00223	0.00455	-0.00366	0.00524	-0.00299
V1165	mtA	*laeta*	25.59	-0.0464	-0.0135	0.00269	-0.00786	-0.0109	0.00288	0.00595	0.0066	0.00152	0.000243	-0.0041
V1166	mtA	*laeta*	26.47	-0.0566	-0.00754	0.0193	-0.00444	-0.0187	0.00443	0.00516	0.00213	0.00287	-0.00376	0.00348
V1167	mtA	*laeta*	23.66	-0.0401	-0.00244	-0.00818	0.0119	-0.0104	-0.0113	-0.00589	0.00604	-0.00216	-0.00866	-0.000968
V1168	mtA	*laeta*	22.52	-0.0187	0.0126	-0.0116	-0.00999	-0.00127	0.00531	0.00139	0.00584	-0.0149	-0.00242	-0.00454
V1169	mtA	*laeta*	24.99	-0.0362	-0.00279	-0.0471	0.00298	-0.0168	-0.00564	-0.0148	0.00711	-0.000455	-0.00119	0.0014
V1170	mtA	*laeta*	23.45	-0.0408	0.0211	-0.00314	-0.0034	-0.00552	-0.00558	-0.00716	0.0108	0.000753	-0.00119	-0.00514
V1171	mtA	*laeta*	25.32	-0.0404	-0.0303	0.00455	-0.0174	-0.0095	-0.0123	-0.00121	0.0113	-0.00346	0.00518	-0.0088
V1172	mtA	*laeta*	25.84	-0.0311	-0.0304	0.00225	-0.00676	-0.0226	0.00326	0.00634	-0.00946	-0.0122	0.0125	-0.00239
V1312	mtA	*laeta*	22.12	0.0205	-0.00614	-0.00565	-0.0127	-0.00608	-0.00202	0.000434	0.00202	0.00253	0.0052	0.0059
V1313	mtA	*laeta*	26.46	0.059	-0.00396	-0.00335	-0.0097	-0.0097	0.00495	-0.00382	0.00082	-0.00349	-0.00854	-0.00643
V1205	mtA	*laeta*	28.15	-0.000646	0.0163	-0.00681	-0.0223	0.00118	-0.00243	0.00822	-0.00343	0.00645	0.000435	-0.00125
V1206	mtA	*laeta*	25.93	-0.0148	-0.011	-0.00333	-0.00271	0.0104	0.0105	0.00522	-0.00147	-0.00452	0.0125	0.0011
V1207	mtA	*laeta*	29.15	-0.00489	-0.00279	-0.00889	-0.00813	-0.00334	0.00115	0.00589	-0.00132	-0.00397	0.00367	0.00694
V1209	mtA	*laeta*	21.35	-0.0541	0.00725	-0.0101	-0.00439	0.00057	-0.00246	-0.00566	-0.00679	0.000889	-0.00655	-0.00591
V1212	mtA	*laeta*	20.94	-0.0363	-0.012	-0.00843	0.0148	0.00251	0.00584	-0.00294	0.00376	-0.00159	-0.000678	-0.00414
V1213	mtA	*laeta*	20.35	-0.0535	0.00544	0.0128	-0.00287	0.00251	-0.00224	0.00385	0.00253	-0.000198	-0.00741	-0.00135
V1215	mtA	*laeta*	20.53	-0.0766	-0.00783	0.0114	0.011	-0.00915	0.0193	-0.00403	0.000736	0.0111	0.0000737	0.00366
V850	mtB	*chinensis*	37.9	0.00165	-0.00664	0.00495	-0.00922	0.00146	-0.00543	-0.000492	0.000172	-0.00194	0.00555	0.00372
V851	mtB	*chinensis*	32.91	0.0257	-0.000453	0.0149	-0.0206	0.00103	-0.00105	0.00138	-0.00498	0.00125	-0.0015	0.00473
V852	mtB	*chinensis*	31.64	0.0105	0.0131	0.0035	-0.0124	-0.0118	0.00511	0.00361	-0.000717	-0.00322	-0.00168	0.00567
V853	mtB	*chinensis*	35.25	0.0299	0.0133	0.00923	-0.0021	-0.00129	-0.00105	0.00165	-0.00388	0.00392	0.00546	0.000233
V1173	mtB	*chinensis*	35.88	0.0444	0.014	0.00912	0.00532	-0.00689	-0.00547	0.00377	-0.00733	-0.00102	-0.00106	-0.00177
V1174	mtB	*chinensis*	32.91	0.0518	0.0128	0.0222	0.00508	-0.0131	-0.0104	0.00539	-0.00326	-0.00395	0.0054	0.00264
V1175	mtB	*chinensis*	36.73	0.0413	0.000586	0.00925	-0.0055	0.000348	-0.00685	-0.0111	0.00133	0.00377	0.00114	0.00594
V1176	mtB	*chinensis*	37.58	0.0602	-0.00849	0.0208	-0.00543	0.00752	0.00158	-0.000677	-0.00424	-0.00188	-0.000758	0.0029
V1177	mtB	*chinensis*	34.22	0.0289	0.00422	0.00517	0.00463	0.0158	-0.00702	-0.00186	0.000801	0.00377	0.00671	-0.00642
V1178	mtB	*chinensis*	25.09	-0.0329	0.0135	0.0186	0.018	0.0132	-0.0146	-0.00108	-0.0107	-0.00441	-0.00467	-0.00125
V1179	mtB	*chinensis*	35.26	0.0375	0.0228	0.00232	0.00592	0.000404	-0.00165	-0.000623	0.00178	-0.000736	0.000812	-0.00292
V1180	mtB	*chinensis*	29.63	-0.0367	-0.000248	0.0165	-0.00205	0.003	-0.00648	-0.00158	0.000867	0.00185	-0.00884	0.00596
V1029	mtC	*chinensis*	24.36	-0.034	0.0278	0.0109	0.00696	0.00982	-0.0045	0.00273	0.00654	0.00651	0.00146	-0.00399
V1030	mtC	*chinensis*	24.17	-0.0312	0.00434	0.00697	0.00476	0.0205	0.00498	-0.00979	0.00225	0.00708	0.00642	-0.00425
V1031	mtC	*chinensis*	27.43	-0.0349	0.0015	0.000186	-0.00829	0.0107	0.00868	-0.00369	-0.00209	-0.00645	-0.00281	-0.00751
V480	mtC	*chinensis*	25.58	0.000683	-0.00153	0.00449	-0.00713	0.0124	0.00162	-0.00252	-0.00131	0.00262	-0.00339	-0.00195
V481	mtC	*chinensis*	30.74	-0.0187	0.0145	-0.00847	-0.00146	0.0057	0.00409	0.00295	0.00384	-0.0114	-0.0015	-0.00637
V705	mtC	*chinensis*	20.19	-0.0687	-0.0069	0.0119	0.0199	-0.00782	0.00771	0.00262	0.00211	0.00887	-0.0019	0.00134
V707	mtC	*chinensis*	25.23	-0.0137	-0.00605	0.00314	-0.000303	0.0218	0.00917	0.000777	-0.000695	-0.0023	0.00148	0.00741
V708	mtC	*chinensis*	24.87	-0.0282	-0.0098	-0.0179	0.0136	0.0131	0.00336	-0.00877	0.00216	0.00254	-0.00252	0.00433
V1065	mtC	*chinensis*	33.32	0.00174	0.033	-0.000494	0.0109	0.00705	-0.00992	-0.00222	0.00336	-0.0039	-0.00364	0.00159
V1066	mtC	*chinensis*	37.19	0.0315	-0.00262	0.00683	-0.00956	-0.00152	-0.00581	-0.0121	-0.00152	-0.000223	-0.00089	0.00269
V1067	mtC	*chinensis*	31.98	0.0169	0.016	0.0186	-0.00407	0.00568	-0.0076	-0.0123	0.00195	-0.00359	0.00486	0.0000862
V1069	mtC	*chinensis*	30.4	0.0125	-0.00511	-0.00442	0.00972	0.0149	-0.0179	0.0311	0.0347	0.00197	0.0078	0.00797
V1070	mtC	*chinensis*	30.08	0.0196	0.0131	0.00991	-0.012	0.0104	-0.00206	-0.00308	-0.00578	0.00846	0.00245	0.00577
V1265	mtC	*chinensis*	23.57	-0.0653	-0.0113	0.0219	-0.00541	0.00622	-0.00472	0.00259	-0.00417	0.00799	-0.00537	-0.00189
V1270	mtC	*chinensis*	22.08	-0.0795	0.0109	0.012	0.00722	-0.000672	-0.00151	0.00772	-0.00384	0.00867	-0.00433	-0.000389
V1271	mtC	*chinensis*	21.32	-0.0538	0.0185	0.00707	0.00174	0.00564	0.00249	-0.00102	-0.000806	0.000193	0.00947	-0.00796
V1272	mtC	*chinensis*	23.41	-0.0721	-0.00847	0.0299	0.0215	-0.00661	-0.00486	-0.00144	-0.00859	0.000915	-0.00989	0.00287
V766	mtD	*chinensis*	33.58	0.0152	0.0226	-0.0184	-0.000168	-0.00886	0.00431	0.000252	-0.00343	0.00747	-0.000747	-0.00132
V767	mtD	*chinensis*	34.7	-0.0215	0.0272	0.000166	-0.00141	0.00921	0.00201	-0.00746	-0.00592	-0.000797	0.00823	-0.00187
V769	mtD	*chinensis*	36.53	0.0408	-0.00372	0.00281	0.0104	0.00349	-0.00935	0.00382	-0.00384	-0.00812	-0.00236	0.00266
V771	mtD	*chinensis*	34.97	0.0637	0.00365	0.0134	0.0082	-0.0156	-0.00623	-0.00394	-0.00385	-0.00191	0.00207	-0.00351
V826	mtD	*chinensis*	25.85	0.00529	-0.0157	-0.00967	-0.0131	-0.00877	-0.0145	-0.0043	0.00851	0.000595	-0.00761	-0.00692
V855	mtD	*chinensis*	36.95	0.0532	-0.0173	0.0049	-0.0023	0.0107	0.00449	-0.00485	0.00397	0.00145	-0.0021	0.00441
V856	mtD	*chinensis*	33.6	0.0618	-0.000805	-0.00152	-0.00142	-0.000933	0.0146	-0.00698	0.00715	-0.002	-0.00883	0.00125
V857	mtD	*chinensis*	36.68	0.0544	-0.00525	-0.0000503	0.00453	0.00607	0.00569	-0.00613	0.0037	-0.00204	-0.00788	-0.00331
V858	mtD	*chinensis*	33.86	0.0354	0.0015	-0.000462	0.00331	0.013	0.00324	-0.00614	0.000793	0.00153	-0.00194	-0.00162
V859	mtD	*chinensis*	34.08	0.069	-0.0072	0.016	0.0123	0.0039	0.0019	-0.00439	0.00279	0.00254	0.00315	-0.0083
V860	mtD	*chinensis*	33.55	0.0667	-0.0114	0.0089	-0.000919	0.00452	0.00645	0.00133	0.00508	0.00166	-0.00368	-0.00134
V861	mtD	*chinensis*	33.06	0.0578	-0.00519	0.0123	0.0124	0.00294	-0.00805	-0.00263	0.00576	-0.00566	-0.00155	0.00543
V862	mtD	*chinensis*	35.77	0.0556	-0.00166	0.00292	0.00881	0.00174	-0.000102	0.00215	-0.000582	0.00378	0.00452	-0.00521
V863	mtD	*chinensis*	32.18	0.0617	-0.00826	0.0126	0.0054	-0.000516	-0.00438	0.00201	0.00384	-0.000279	-0.00403	0.00234
V864	mtD	*chinensis*	33.7	0.0684	-0.0141	0.0071	0.00845	-0.00165	0.00179	-0.000966	-0.000256	0.000127	-0.00225	-0.0035
V865	mtD	*chinensis*	36.95	0.0489	0.00948	-0.00701	0.000759	-0.0055	0.00137	-0.00671	0.00875	0.00244	-0.00623	0.00275
V866	mtD	*chinensis*	33.65	0.0645	0.0108	0.00283	0.0175	-0.00073	0.00884	-0.000991	0.000476	0.00276	0.00622	-0.00569
V867	mtD	*chinensis*	38.92	0.0823	-0.00606	0.0155	0.000952	-0.00487	0.0144	-0.00439	0.00514	-0.00377	0.00129	-0.000259
V868	mtD	*chinensis*	36.38	0.0356	0.00903	-0.00248	-0.00186	-0.000985	0.00649	-0.00489	0.00416	0.00574	0.00106	0.00504
V869	mtD	*chinensis*	36.21	0.061	-0.00366	0.01	-0.00421	0.00879	0.00289	-0.00838	0.00584	-0.00408	-0.00558	-0.00301
V870	mtD	*chinensis*	40.88	0.039	-0.0118	0.000765	0.00171	0.0105	0.00364	0.00706	0.0265	0.00318	-0.00805	0.00155
V871	mtD	*chinensis*	35.22	0.084	-0.0101	0.0149	0.00768	-0.0133	0.00647	-0.000715	0.00324	-0.00477	0.00278	-0.00169
V872	mtD	*chinensis*	39.3	0.0401	0.00896	0.0049	0.00494	0.00887	0.00699	-0.00525	-0.000556	0.00242	-0.00442	-0.00105
V873	mtD	*chinensis*	36.18	0.0449	0.0148	0.00494	-0.000726	0.0071	0.00136	-0.000329	0.0056	-0.00782	-0.0127	-0.00165
V874	mtD	*chinensis*	35.7	0.0502	0.0157	0.00205	0.0173	-0.00567	0.00555	0.00561	0.0057	-0.00413	-0.000659	0.000602
V875	mtD	*chinensis*	37.04	0.0725	0.01	0.00293	0.0177	-0.00841	0.0135	-0.00174	-0.000185	-0.00456	0.000237	0.000915
V1045	mtD	*chinensis*	28.66	0.015	0.0221	-0.00718	-0.00367	-0.00583	0.00926	-0.00571	0.00962	-0.00806	-0.00189	0.00674
V1046	mtD	*chinensis*	29.69	-0.0223	0.0187	-0.0111	0.00141	-0.00811	-0.0134	-0.00796	-0.00191	-0.00495	-0.00817	0.00243
V1047	mtD	*chinensis*	28.18	0.00342	0.0245	-0.0103	-0.00163	-0.00426	0.00058	-0.00315	-0.000539	-0.00585	-0.0031	0.000118
V1048	mtD	*chinensis*	26.51	0.0146	-0.00467	0.00553	-0.024	-0.000414	-0.00113	-0.00642	-0.00148	0.00869	0.00066	-0.000754
V1049	mtD	*chinensis*	26.19	-0.0357	0.00033	-0.00622	-0.00499	-0.00167	-0.0167	-0.0129	-0.00469	0.0015	0.00453	-0.00771
V1050	mtD	*chinensis*	25.64	-0.000621	-0.00946	-0.00169	-0.0097	0.00447	-0.00818	-0.00157	0.00196	0.00731	-0.000592	0.0101
V1051	mtD	*chinensis*	25.03	0.0114	-0.0117	0.0101	-0.0177	0.0144	-0.00248	-0.00258	0.000278	0.00679	-0.00219	0.00507
V1052	mtD	*chinensis*	25.89	-0.0256	-0.0013	0.000966	-0.0111	0.000366	0.00179	0.000996	0.00557	-0.00801	0.00268	-0.00812
V1053	mtD	*chinensis*	25.92	-0.00764	-0.00838	0.00537	0.00125	-0.000223	-0.00577	0.0000475	-0.000523	-0.00546	0.00392	0.00257
V1054	mtD	*chinensis*	24.63	-0.0204	0.00875	0.00155	-0.00754	0.011	0.00101	-0.00427	-0.0049	-0.00339	-0.00133	-0.00776
V1084	mtD	*chinensis*	37.57	0.0168	-0.00122	0.00212	-0.0142	-0.00399	-0.00241	-0.00497	0.00716	-0.00363	0.00382	0.00875
V1086	mtD	*chinensis*	26.14	-0.0133	0.0109	0.00577	-0.00705	-0.00271	-0.0102	0.00378	-0.00253	-0.00361	0.00135	0.00569
V1087	mtD	*chinensis*	25.16	-0.0385	0.0181	-0.00287	0.00442	0.00527	0.0114	-0.00573	0.00928	-0.0000995	0.0095	-0.00274
V1088	mtD	*chinensis*	24.6	-0.0481	-0.00272	-0.000664	0.00458	-0.0122	-0.00638	-0.0176	0.00378	0.00667	-0.00478	-0.00058
V1089	mtD	*chinensis*	24.26	0.0395	0.0226	-0.00863	0.0129	0.0142	0.0104	-0.00551	0.000943	0.00354	0.00209	0.000399
V1090	mtD	*chinensis*	25.46	-0.0023	0.00162	0.00604	0.00152	0.00717	-0.00511	0.00233	-0.00384	-0.00111	0.0115	0.0051
V1091	mtD	*chinensis*	26.93	-0.0258	-0.00179	-0.00144	0.000267	-0.00348	-0.00713	-0.00719	0.00869	0.000236	-0.000818	-0.00249
V1092	mtD	*chinensis*	28.26	-0.0102	-0.00686	-0.00449	-0.00675	0.00125	-0.00862	-0.0229	0.00366	0.00628	0.00758	0.00253
V1093	mtD	*chinensis*	25.9	0.0118	0.00943	-0.00778	-0.004	-0.000464	-0.00246	-0.00417	0.00566	0.00505	0.00411	0.00838
V1094	mtD	*chinensis*	26.01	-0.0581	0.00241	0.00623	0.00188	-0.00554	0.0127	-0.00902	0.00559	0.0114	0.00495	-0.0059
V1095	mtD	*chinensis*	27.53	-0.0339	-0.0121	0.00921	0.00717	-0.00396	-0.0168	0.00752	0.0118	0.000569	0.00022	-0.00981
V1096	mtD	*chinensis*	24.71	-0.055	0.00297	0.00906	0.00224	-0.00477	0.00579	-0.00812	0.00408	0.00934	0.00193	0.00146
V1097	mtD	*chinensis*	24.35	-0.0269	-0.0235	0.00219	-0.00414	0.0113	-0.00884	-0.0131	0.00154	0.00702	0.0000707	-0.000991
V1098	mtD	*chinensis*	21.86	-0.0483	0.012	0.0176	0.0147	0.00273	-0.0174	-0.00496	-0.00787	0.0018	0.00321	-0.000125
V1368	mtD	*chinensis*	42.86	0.0538	0.000239	0.0113	0.0139	-0.0148	-0.00369	0.000376	-0.00789	-0.00349	0.00156	0.00185

Table S6. Summary of canonical structures for the morphological analysis of shells.

Parameter	CDA (mtDNA) CAN 1	CAN 2	CDA (nDNA) CAN 1
Eigenvalue	1.445	0.235	1.429
Cum. Prop. (%)	77.58	90.21	100.00
Coefficient			
D	0.203	0.455	0.174
PC1	0.376	0.641	0.346
PC2	0.307	-0.228	0.311
PC3	0.528	-0.322	0.529
PC4	0.156	-0.097	0.166
PC5	0.250	-0.524	0.273
PC6	-0.184	0.126	-0.179
PC7	-0.490	-0.299	-0.486
PC8	0.332	0.115	0.342
PC9	0.104	-0.153	0.113
PC10	-0.018	-0.017	-0.020
PC11	0.043	-0.058	0.038

Table S7. Information on sequence alignments and models of sequence evolution for maximum likelihood and Bayesian analysis.

Alignment	Length of alignment	Excluded sites	Model of sequence evolution	
			ML	Bayes
COI	404	-	GTR+Gamma	position 1: K80+Gamma / position 2: HKY / position 3: HKY+Gamma
16S	384	159-161, 168, 191, 192, 255, 266, 270, 271	GTR+Gamma	HKY+Gamma

Table S8. Information on parameter settings used in DIYABC.

Parameter	Minimum	Maximum
Effective population size		
N1	10	500000
N2	10	100000
N3	10	500000
NA	10	5000000
Time scale in generations		
t1	60	300000
t2	60	10000000
Admixture		
ra	0.001	0.999

Time scale in generations were set t2>t1 condition.

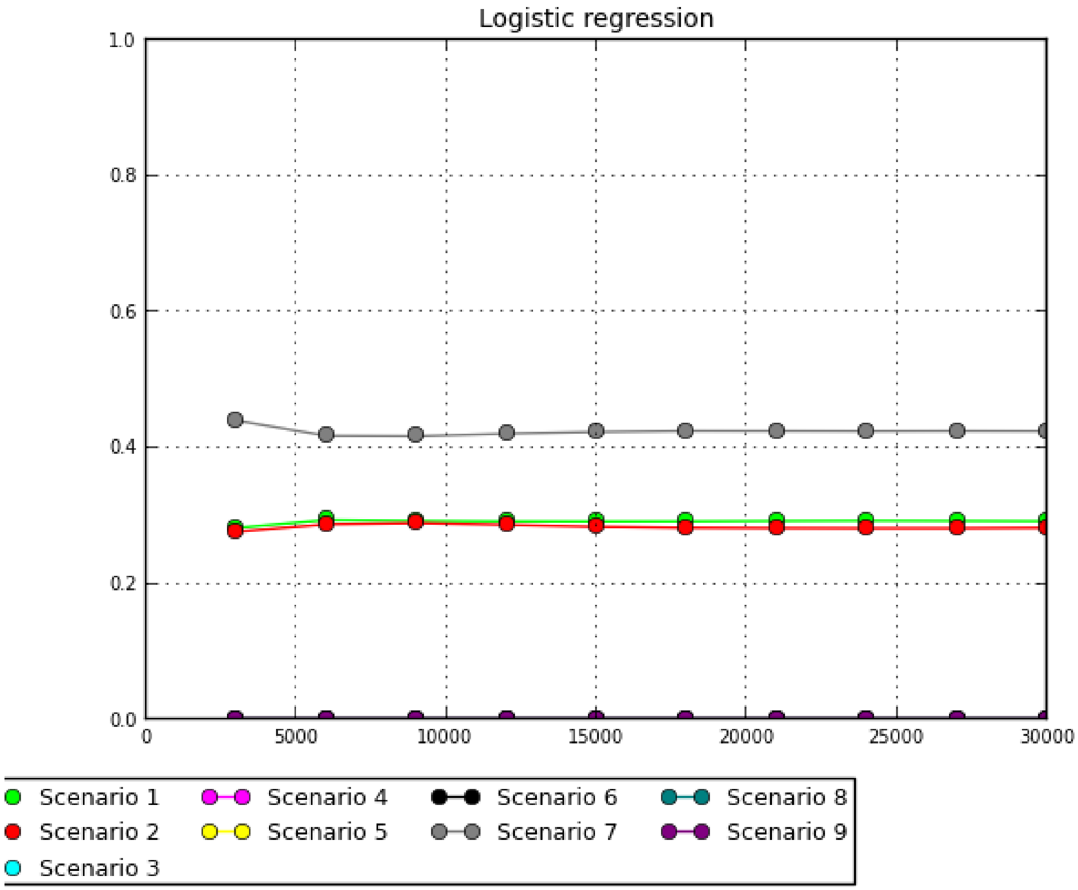

Figure S1. Result of the DIYABC analysis Model comparison using the logistic regression approach.

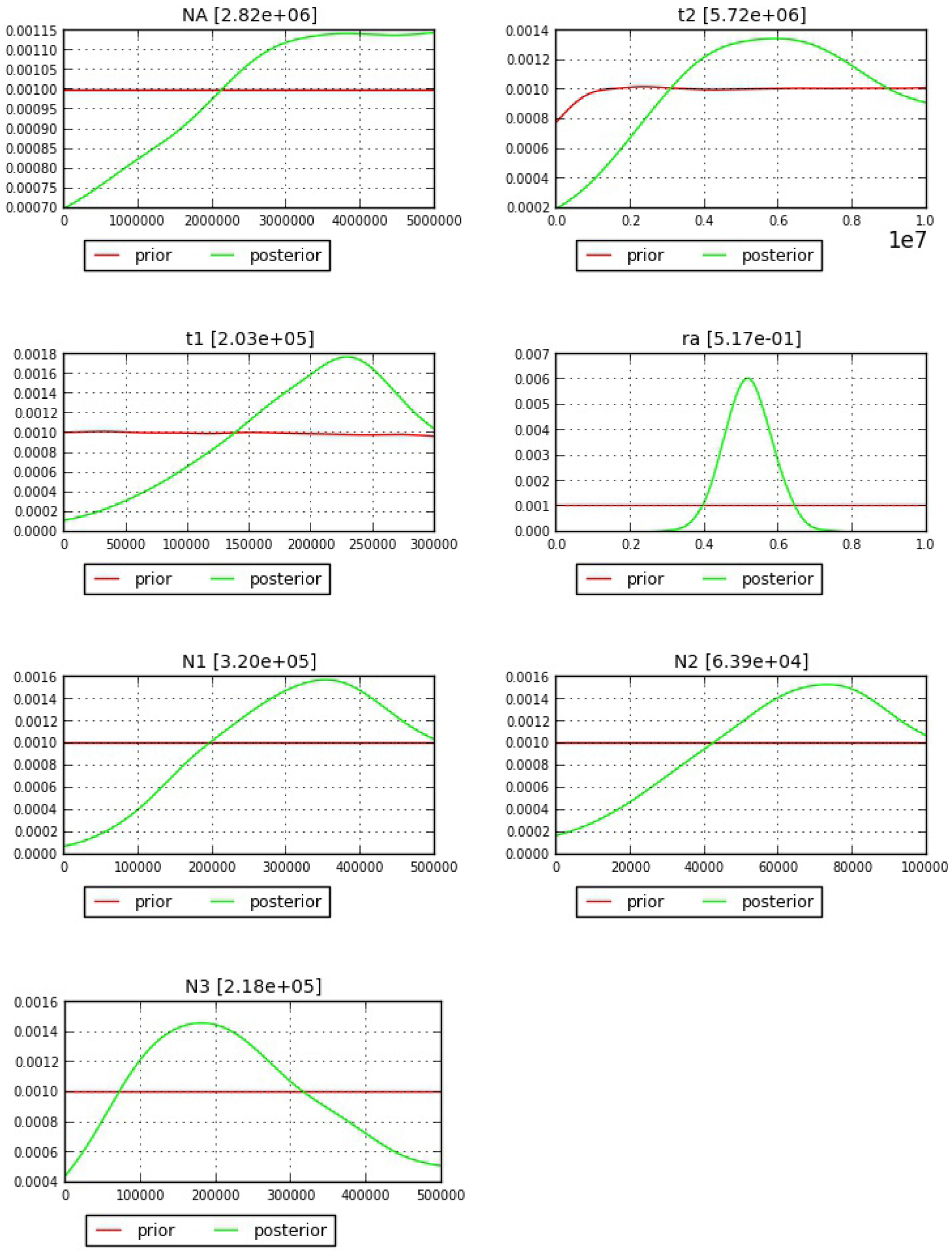

Figure S2. The prior and posterior distributions for each parameter obtained using DIYABC. The Y axis represents the probability densities of priors and posteriors.

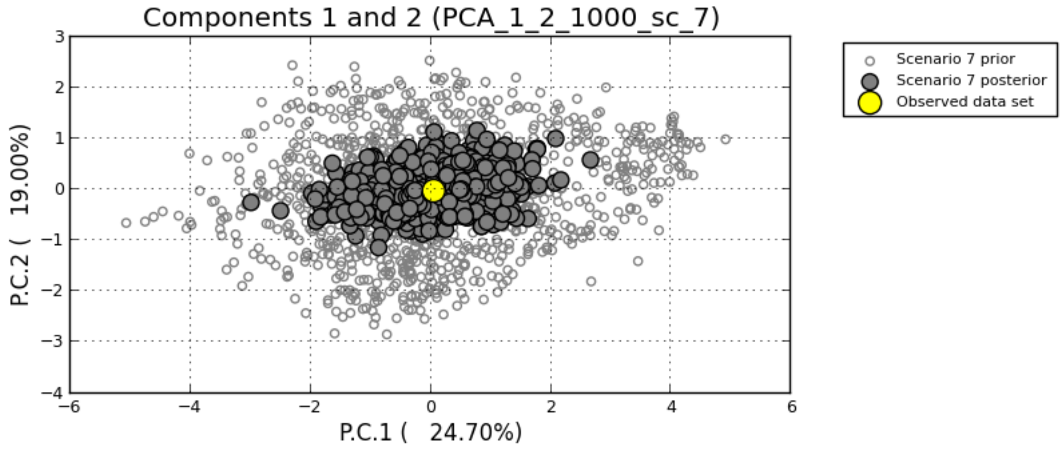

Figure S3. The result of PCA from data set on DIYABC model checking option.
